# Hypertension prevalence, detection, treatment and control among 1.4 million adults in England: a cross-sectional analysis from the Our Future Health study in 2022–2025

**DOI:** 10.1136/bmjph-2025-004815

**Published:** 2026-09-23

**Authors:** Wenyu Liu, Jennifer Collister, Thomas Littlejohns, Iain James Turnbull, Robert Clarke, David John Hunter

**Affiliations:** 1 Nuffield Department of Population Health, Big Data Institute, University of Oxford, Oxford, UK; 2 School of Public Health Department of Epidemiology, Harvard T H Chan, Boston, Massachusetts, USA

**Keywords:** Hypertension, Public Health, Epidemiology, Demography, Prevalence

## Abstract

**Objectives:**

To assess the prevalence, detection, treatment and control of hypertension in England.

**Design:**

Cross-sectional analysis of baseline blood pressure measurements from the Our Future Health study.

**Setting:**

Over 200 enrolment centres across England, 2022–2025.

**Participants:**

1.4 million adults aged ≥18 years with available clinic blood pressure measurements.

**Main outcome measures:**

Hypertension was defined according to WHO guidelines as systolic blood pressure (SBP) ≥140 mm Hg, diastolic blood pressure (DBP) ≥90 mm Hg or self-reported use of antihypertensive medication. Detection was defined as self-reported prior diagnosis by a health professional or reported use of antihypertensive medication. Controlled hypertension was defined as SBP <140 mm Hg and DBP <90 mm Hg among those receiving antihypertensive treatment.

**Results:**

Overall, 37.2% of participants had hypertension, with higher age-standardised prevalence in men than women (41.9% vs 28.6%). Prevalence increased markedly with age, from 23% to 73% in men and from 10% to 69% in women at ages 18–39 and ≥80 years, respectively. Despite this high burden, 58.8% of individuals with hypertension were undetected. Hypertension was strongly associated with older age, male sex and higher body mass index, but the prevalence remained substantial across all population subgroups. Factors associated with hypertension detection largely mirrored those associated with hypertension, except in individuals with prior cardiovascular disease or diabetes, among whom detection was higher. Among detected participants, antihypertensive treatment was associated with lower odds of uncontrolled blood pressure; however, 50.2% of treated individuals did not achieve blood pressure control.

**Conclusions:**

Over one-third of adults in England had hypertension, yet only half of affected individuals were detected and half of those treated achieved blood pressure control. The high prevalence across nearly all population subgroups underscores the need for population-wide prevention strategies alongside targeted treatment of individuals at high cardiovascular risk.

**WHAT IS ALREADY KNOWN ON THIS TOPIC**

- Information on hypertension in England is limited to data in medical records as well as relatively small surveys, such as the Office of National Statistics Health and Lifestyle Surveys, and the National Health Service Health Check programme that starts at age 40.

**WHAT THIS STUDY ADDS**

- In a cross-sectional survey of 1.4 million adult participants 18 year and older from the Our Future Health study in 2022–2025, 37% of participants had a blood pressure of >=140/90 mm Hg or reported taking one or more antihypertensive medications (42% in men, 29% in women). As expected, the prevalence of hypertension increased with age, was higher in men than women, and increased with body mass index; however, the prevalence of hypertension was substantial in all demographic groups.
- Less than half of all participants with hypertension were detected and less than half of those treated with antihypertensive medications had their blood pressure controlled.

**HOW THIS STUDY MIGHT AFFECT RESEARCH, PRACTICE OR POLICY**

- High blood pressure needs to be considered as a population-wide problem in England and new approaches to diagnosis and treatment considered.
- The current guidelines that advocate restricting initiation of antihypertensive medication to individuals with confirmed diagnoses using multiple home measurements or ambulatory blood pressure monitoring should be reconsidered to address the high levels of uncontrolled high blood pressure in England.

## Introduction

 High systolic blood pressure (SBP) is a leading cause of morbidity and mortality in the UK and worldwide.^1,2^ Hypertension affects approximately one-third of adults aged 30–79 years globally.^3^ Contemporary population-based evidence on the prevalence and determinants of hypertension, including levels of detection, treatment and control of hypertension in the UK, is limited. The Office of National Statistics (ONS) Health and Lifestyle Surveys typically record blood pressure (BP) in about 4000 households annually from a larger number of randomly sampled addresses.^4^ The Clinical Practice Research Datalink and the Cardiovascular Disease Prevention Audit record BP in patients attending General Practices in the UK.^5,6^ In an analysis of secondary care records to determine the prevalence of long-term medical conditions in the UK, hypertension was the most commonly diagnosed condition in adults aged 50 years or older.^7^ The National Health Service (NHS) ‘Health Check’ also collects data on population-wide prevalence of hypertension in adults aged 40–74 years but provides limited data on risk factors.^8^

The Our Future Health (OFH) study provides contemporary large-scale evidence on clinically measured BP among adults aged ≥18 years who were living in the UK. Data are currently available on 1 428 494 adults who attended an enrolment clinic visit in England between 2022 and 2025, had their BP measured together with standardised weight and height measurements, and completed a questionnaire providing details on demographic factors and lifestyle risk factors and health conditions. This report examines the prevalence, detection, treatment and control of hypertension in a contemporary large study in the English population, and also assesses associations of risk factors for diagnosis, detection, treatment and control of hypertension.

## Methods

OFH is a prospective cohort study of a representative sample of all UK-resident adults.^9^ Invitation letters were mailed to all potentially eligible individuals or households, typically living within a 5 mile radius, prior to opening a new in-person enrolment centre using personalised letters sent via NHS DigiTrials (https://digital.nhs.uk/services/nhs-digitrials) or non-personalised letters to households that included an invitation to join the programme and a QR code and web address to provide details about the study. Recruitment was initially focused on English regions with high socioeconomic deprivation and ethnic diversity (West Midlands, Greater Manchester, London and West Yorkshire) to increase opportunities for participation among historically under-represented groups.^10^ Awareness and engagement were supported by advertisements in local and national media and by outreach to local community stakeholders. Consenting participants completed a baseline questionnaire that recorded information on personal demographics and household, work and education, lifestyle exposures, family health history and personal health history (available in the Study Protocol Appendix at https://research.ourfuturehealth.org.uk/). At an in-person enrolment centre visit, anthropometric and BP measurements were recorded. While this specific study did not involve patients or public contributors directly in its design or conduct, the OFH research infrastructure integrates public involvement at multiple levels. The Access Board (https://ourfuturehealth.org.uk/about-us/how-were-governed/the-access-board/), which reviews all applications for data access, includes members of the public and participant representatives alongside scientific experts, providing participant-centred perspectives in governance. In addition, the programme supports a broader Public Advisory Board and Involvement Network that contributes to shaping research priorities and frameworks within the overall programme.

Measurement of BP: BP was measured using an automated Omron 3 or Omron HBP 1320 device. Participants sat with their feet flat on the ground, toes pointing forward and their back upright and the fully deflated cuff of the appropriate size snugly around the participant’s upper arm ensuring that the arm was level with their heart. BP was recorded twice and on a third occasion if the first or second SBP readings were both ≥160 mm Hg or <90 mm Hg. If there were more than two measurements, we report the average of the second and third measurements. If participants had average BP measurements between 140/90 and 179/109 mm Hg, they were asked to seek repeat measurements at their local pharmacy within the next few weeks, but if they had BP readings ≥180/110 mm Hg, they were asked to seek a repeat measurement at their general practitioner (GP) or pharmacy within the next 2 days.

Definitions of hypertension: ‘Hypertension’ was defined based on the WHO guidelines as the number of participants who (1) had mean SBP ≥140 mm Hg or mean diastolic BP (DBP) of ≥90 mm Hg or (2) were currently taking an antihypertensive medication.^3^ Hypertensive participants were further divided into ‘undetected’, ‘detected, not treated’, ‘treated, not controlled’ and ‘controlled’. ‘Detection’ of hypertension was defined if they reported a prior diagnosis of hypertension by a health professional or were receiving BP lowering medication, among individuals who were hypertensive. ‘Treatment’ was defined if participants reported use of antihypertensive medications. Uncontrolled hypertension was defined among those treated as still having a mean SBP of at least 140 mm Hg or a mean DBP of at least 90 mm Hg. Details of the mapping of these definitions to the questionnaires are provided in the online supplemental methods.

Risk factors for hypertension: Information was collected on age, sex, (self-assigned) ethnicity, body mass index and season of enrolment (from the date of registration), household income, education, smoking, alcohol consumption, family history of cardiovascular disease (CVD), personal history of CVD or diabetes, and frequency of falls (from the questionnaire). Additional details on these variables are provided in the online supplemental appendix.

### Statistical methods

We excluded a small number (0.03%) of participants with implausible BP measurements (SBP <70 or ≥270 mm Hg; DBP <50 or ≥150 mm Hg), and a larger number (8.1%) who reported serious health conditions (including kidney failure, heart failure, liver failure and cancer other than basal/squamous cell skin cancer) since conditions associated with a poor prognosis often affect BP and mandate treatment of hypertension, which may thus distort the prevalence of hypertension statistics. In the supplement, we provide analyses including these participants. We calculated the prevalence of hypertension by sex and age. In addition, age-standardised and sex-standardised prevalence were obtained using direct standardisation, with the ONS England population age and sex distribution estimates in mid-2023 considered the standard population. We compared the prevalence of hypertension based on the WHO definitions with prevalent hypertension based on alternative guidelines and the prevalence of self-report/no self-report of hypertension diagnosis, respectively.

We used logistic regression to conduct multivariate analyses of the above-mentioned risk factors for hypertension. Similarly, logistic regression models were performed to evaluate the associations with hypertension detection and baseline characteristics, adjusted for age and sex and additional confounders. We investigated the associations between individual types of comorbidities (namely, CVD, arrhythmia, diabetes, asthma or chronic obstructive pulmonary disease, migraines, epilepsy, anxiety, depression, osteoarthritis, other joint disorder) and (1) hypertension and (2) detection, respectively; these comorbidities may increase physicians’ index of suspicion that hypertension is a clinically relevant condition. We conducted sensitivity analyses at different thresholds of measured BP among participants without reporting BP-lowering medication. Among the study population with detected hypertension, we investigated risk factors associated with hypertension. Finally, we examined the associations of use of one or more antihypertensive medications with a history of falls since medications that lower BP or slow the heart rate can cause falls by reducing blood flow to the brain, leading to fainting or dizziness.

## Results

### Prevalence of hypertension

Table 1 shows baseline characteristics of the study population, with and without hypertension defined using the WHO criteria. In total, 115 531 participants (8.1%) had serious health conditions (online supplemental figure S1), including 61 886 and 53 645 with and without hypertension, respectively. Among 1 312 551 participants with no prior serious medical conditions, 37.2% had hypertension and the prevalence was greater in men than women. The prevalence of hypertension increased with age (from 23% to 73% in men and 10% to 69% in women at age 18–39 to 80+ years, respectively), but about 58.8% of those with hypertension were undetected (figure 1 and online supplemental figure S1). Among those with no self-report of hypertension diagnosis, 33.2% of men and 21.2% of women had a BP ≥140/90 mm Hg and a similar proportion had BP ≥130/80 but <140/90 mm Hg (online supplemental table S1). Among those who reported taking one or more antihypertensive medications (n=182 876), 50.2% still had a BP ≥140/90 mm Hg (online supplemental figure S1). When age-standardised to the UK population, 41.9% of men and 28.6% of women had hypertension. Similar estimates of the prevalence of hypertension were obtained using alternative diagnostic criteria that considered self-reported diagnosis of hypertension (instead of use of antihypertensive medications) in addition to elevated BP (online supplemental table S1).

**Table 1:** Baseline characteristics of the study population, with and without hypertension (HTN) defined using WHO criteria

Characteristics	No HTN	With HTN	All
	(n=823 822)	(n=488 729)	(n=1 312 551)
Age, years			
18–39	264 252 (32.1%)	47 656 (9.8%)	311 908 (23.8%)
40–49	157 001 (19.1%)	53 864 (11.0%)	210 865 (16.1%)
50–59	176 050 (21.4%)	108 633 (22.2%)	284 683 (21.7%)
60–69	154 715 (18.8%)	153 913 (31.5%)	308 628 (23.5%)
70–79	64 307 (7.8%)	106 269 (21.7%)	170 576 (13.0%)
80+	7452 (0.9%)	18 283 (3.7%)	25 735 (2.0%)
Missing	45 (0.0%)	111 (0.0%)	156 (0.0%)
Sex*			
Female	517 603 (62.8%)	214 571 (43.9%)	732 174 (55.8%)
Male	305 709 (37.1%)	273 902 (56.0%)	579 611 (44.2%)
BMI†			
Underweight/normal	380 795 (46.2%)	110 712 (22.7%)	491 507 (37.4%)
Overweight	299 847 (36.4%)	197 522 (40.4%)	497 369 (37.9%)
Obese	141 663 (17.2%)	179 535 (36.7%)	321 198 (24.5%)
Missing	1517 (0.2%)	960 (0.2%)	2477 (0.2%)
Ethnicity‡			
White	722 766 (87.7%)	450 322 (92.1%)	1 173 088 (89.4%)
Black	13 169 (1.6%)	8204 (1.7%)	21 373 (1.6%)
South Asian	34 215 (4.2%)	12 739 (2.6%)	46 954 (3.6%)
East Asian	12 291 (1.5%)	3556 (0.7%)	15 847 (1.2%)
Other	41 380 (5.0%)	13 908 (2.8%)	55 288 (4.2%)
Household income, GBP			
>100 000	126 063 (15.3%)	45 167 (9.2%)	171 230 (13.0%)
52 000–99 999	236 930 (28.8%)	111 621 (22.8%)	348 551 (26.6%)
31 000–51 999	161 712 (19.6%)	108 474 (22.2%)	270 186 (20.6%)
18 000–30 999	107 826 (13.1%)	88 030 (18.0%)	195 856 (14.9%)
<18 000	64 987 (7.9%)	51 672 (10.6%)	116 659 (8.9%)
Missing	126 304 (15.3%)	83 765 (17.1%)	210 069 (16.0%)
Education§			
Tertiary	572 148 (69.5%)	288 084 (58.9%)	860 232 (65.5%)
Postsecondary non-tertiary	42 492 (5.2%)	37 480 (7.7%)	79 972 (6.1%)
Secondary	170 236 (20.7%)	122 550 (25.1%)	292 786 (22.3%)
None of the above	27 443 (3.3%)	32 751 (6.7%)	60 194 (4.6%)
Missing	11 503 (1.4%)	7864 (1.6%)	19 367 (1.5%)
Smoking status			
Never smoker	544 807 (66.1%)	291 183 (59.6%)	835 990 (63.7%)
Former smoker	204 290 (24.8%)	158 776 (32.5%)	363 066 (27.7%)
Current smoker	53 221 (6.5%)	27 035 (5.5%)	80 256 (6.1%)
Missing	21 504 (2.6%)	11 735 (2.4%)	33 239 (2.5%)
Alcohol status			
Never drinker	47 249 (5.7%)	20 782 (4.3%)	68 031 (5.2%)
Former drinker	42 164 (5.1%)	24 331 (5.0%)	66 495 (5.1%)
Current drinker	731 247 (88.8%)	441 912 (90.4%)	1 173 159 (89.4%)
Missing	3162 (0.4%)	1704 (0.3%)	4866 (0.4%)
Family history of CVD			
No	556 885 (67.6%)	263 424 (53.9%)	820 309 (62.5%)
Yes	266 937 (32.4%)	225 305 (46.1%)	492 242 (37.5%)
Prior CVD			
No	812 463 (98.6%)	446 542 (91.4%)	1 259 005 (95.9%)
Yes	11 359 (1.4%)	42 187 (8.6%)	53 546 (4.1%)
Diabetes			
No	811 026 (98.4%)	465 625 (95.3%)	1 276 651 (97.3%)
Yes	12 796 (1.6%)	23 104 (4.7%)	35 900 (2.7%)
Frequency of fall¶			
0	704 758 (85.5%)	400 197 (81.9%)	1 104 955 (84.2%)
1	77 421 (9.4%)	56 277 (11.5%)	133 698 (10.2%)
>2	37 361 (4.5%)	30 232 (6.2%)	67 593 (5.1%)
Missing	4282 (0.5%)	2023 (0.4%)	6305 (0.5%)
Season at the time of enrolment**			
Winter	201 037 (24.4%)	127 526 (26.1%)	328 563 (25.0%)
Spring	219 568 (26.7%)	124 721 (25.5%)	344 289 (26.2%)
Summer	203 792 (24.7%)	111 660 (22.8%)	315 452 (24.0%)
Autumn	199 425 (24.2%)	124 822 (25.5%)	324 247 (24.7%)
Number of blood pressure lowering medications††			
0	803 704 (97.6%)	296 595 (60.7%)	1 100 299 (83.8%)
1	NA	142 714 (29.2%)	142 714 (10.9%)
2	NA	30 135 (6.2%)	30 135 (2.3%)
>3	NA	4243 (0.9%)	4243 (0.3%)
Missing	20 118 (2.4%)	15 042 (3.1%)	35 160 (2.7%)

* ‘Other/Intersex’ and ‘Prefer not to answer’ were omitted here due to small percentages (<0.1%).

† Underweight: <18.5 kg/m2; normal: 18.5–24.9; overweight: 25–29.9; obese: ≥30.

‡ There was only one male participant with missing ethnicity data (omitted here).

§ Highest level of education was based on International Standard Classification of Education.

¶ Frequency of falls (not during playing sports or exercise) in the last year.

** Winter: December–February; Spring: March–May; Summer: June–August; Autumn: September–November.

†† Blood pressure lowering medications include calcium channel blocker, blood pressure medication, beta blocker and diuretic. Note that these data were available from participants who completed Version 2 questionnaire only. Version 1 of the questionnaire, which was completed by approximately 52 000 participants, had a different and smaller set of options for the health history questions.

BMI, body mass index; CVD, cardiovascular disease; NA, not available.

**Figure 1:**
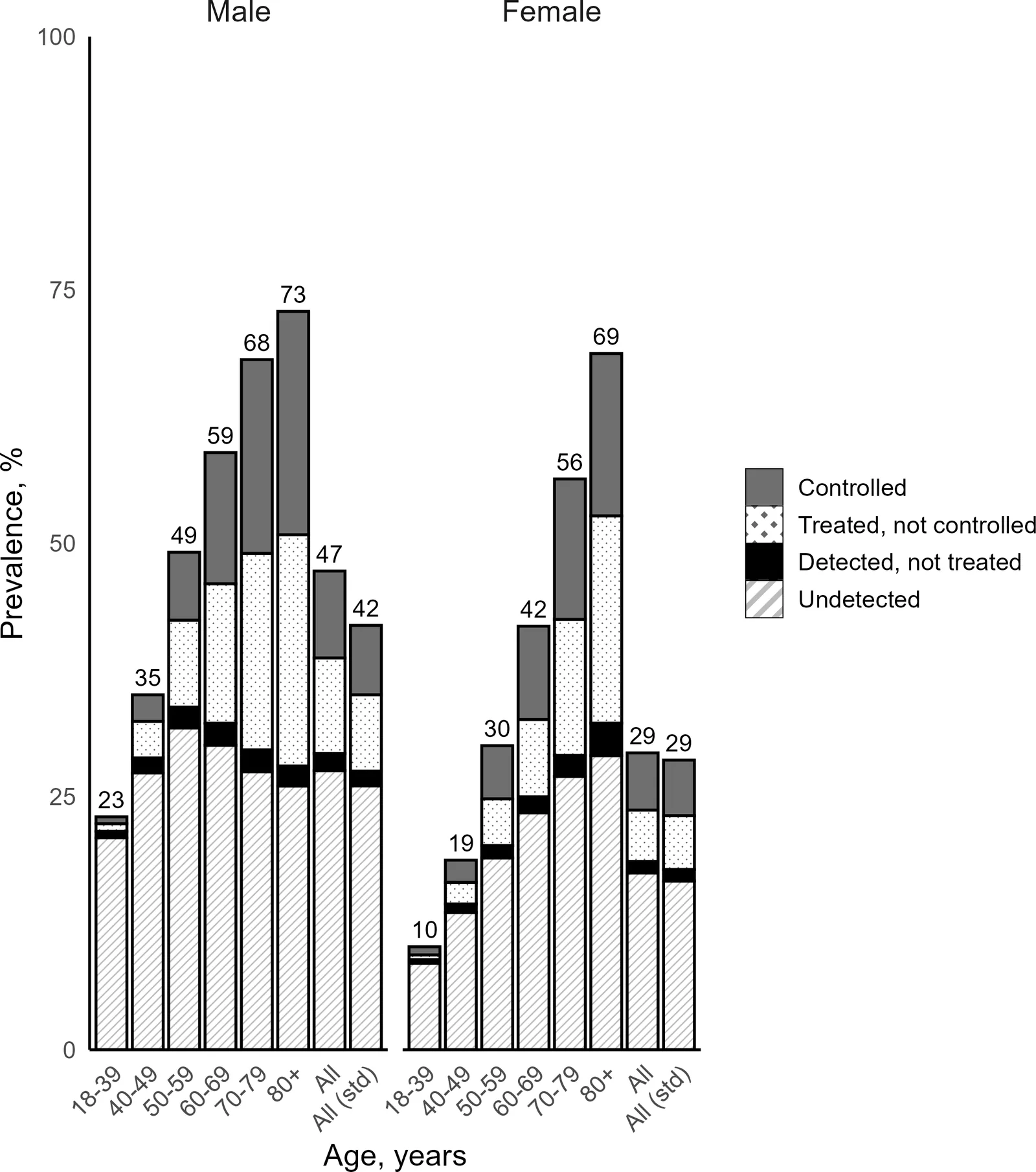
Prevalence of hypertension by age and sex, defined using WHO criteria, with crude prevalence for all age groups by sex denoted as ‘All’ and age-standardised prevalence as ‘All (std)’. Direct standardisation was based on the Office for National Statistics population estimates for England in mid-2023.

### Risk factors for hypertension

As expected, the prevalence of hypertension increased with age, however, 15.3% of participants aged 18–39 years had a BP ≥140/90 mm Hg or self-report of receiving BP lowering medication. Men were approximately twice as likely to have hypertension in both age-adjusted and multivariate analyses (table 2, figure 2). The odds of hypertension were about 50% higher in Black than in White participants, but these differences were attenuated to about 30% higher in the multivariate analyses. The prevalence of hypertension was lower among South Asians and East Asians compared with White British, although these differences were also attenuated in the multivariate analyses. In the fully adjusted models, overweight and obesity were monotonically associated with hypertension, in addition to past and current smoking and current alcohol consumption. Years spent in education and higher income were only weakly associated with hypertension. Both a family history and a personal history of CVD or diabetes were associated with higher risk of hypertension. The odds of hypertension were lower in Spring and Summer than in the Winter and Autumn (table 2). Analyses including those with serious health conditions were similar to the above observations (online supplemental figure S2 and S3 and table S10).

**Table 2:** Associations of risk factors with hypertension defined using WHO criteria (Yes vs No (reference)) in the study population (N=1 312 551)

			Age and sex adjusted*			Multivariable adjusted†	
Covariates	N	Hypertension, %	OR (95% CI)		P value	OR (95%CI CI)	P value
Age, year**‡**							
18–39	311 908	15.3	1 (ref)		–	1 (ref)	–
40–49	210 865	25.5	1.90 (1.87 to 1.93)		<0.001	1.75 (1.73 to 1.78)	<0.001
50–59	284 683	38.2	3.46 (3.42 to 3.50)		<0.001	2.92 (2.88 to 2.96)	<0.001
60–69	308 628	49.9	5.48 (5.42 to 5.55)		<0.001	4.52 (4.46 to 4.58)	<0.001
70–79	170 576	62.3	9.02 (8.89 to 9.14)		<0.001	7.69 (7.57 to 7.81)	<0.001
80+	25 735	71	13.02 (12.65 to 13.40)		<0.001	11.57 (11.22 to 11.94)	<0.001
Sex**‡**							
Female	732 174	29.3	1 (ref)		–	1 (ref)	–
Male	579 611	47.3	2.11 (2.10 to 2.13)		<0.001	2.06 (2.04 to 2.08)	<0.001
BMI**‡**							
Underweight/normal	491 507	22.5	1 (ref)		–	1 (ref)	–
Overweight	497 369	39.7	1.94 (1.92 to 1.96)		<0.001	1.90 (1.88 to 1.92)	<0.001
Obese	321 198	55.9	4.82 (4.77 to 4.87)		<0.001	4.55 (4.50 to 4.60)	<0.001
Ethnicity							
White	1 173 088	38.4	1 (ref)		–	1 (ref)	–
Black	21 373	38.4	1.54 (1.49 to 1.58)		<0.001	1.31 (1.27 to 1.35)	<0.001
South Asian	46 954	27.1	0.87 (0.85 to 0.89)		<0.001	0.93 (0.90 to 0.95)	<0.001
East Asian	15 847	22.4	0.66 (0.63 to 0.68)		<0.001	1.00 (0.96 to 1.05)	0.87
Other	55 288	25.2	0.83 (0.82 to 0.85)		<0.001	0.86 (0.84 to 0.88)	<0.001
Household income, GBP							
>100 000	171 230	26.4	0.68 (0.67 to 0.69)		<0.001	0.79 (0.78 to 0.80)	<0.001
52 000–100 000	348 551	32.0	0.88 (0.87 to 0.89)		<0.001	0.92 (0.91 to 0.93)	<0.001
31 000–51 999	270 186	40.1	1 (ref)		–	1 (ref)	–
18 000–30 999	195 856	44.9	1.08 (1.07 to 1.10)		<0.001	1.03 (1.02 to 1.04)	<0.001
<18 000	116 659	44.3	1.12 (1.10 to 1.13)		<0.001	1.00 (0.99 to 1.02)	0.77
Missing	210 069	39.9	0.99 (0.98 to 1.00)		0.16	0.99 (0.98 to 1.00)	0.11
Education**‡**							
Tertiary	860 232	33.5	1 (ref)		–	1 (ref)	–
Post-secondary non-tertiary	79 972	46.9	1.18 (1.16 to 1.20)		<0.001	1.09 (1.08 to 1.11)	<0.001
Secondary	292 786	41.9	1.22 (1.21 to 1.24)		<0.001	1.12 (1.11 to 1.13)	<0.001
None of the above	60 194	54.4	1.39 (1.36 to 1.41)		<0.001	1.15 (1.12 to 1.17)	<0.001
Smoking status**‡**							
Never smoker	835 990	34.8	1 (ref)		–	1 (ref)	–
Former smoker	363 066	43.7	1.15 (1.14 to 1.16)		<0.001	1.02 (1.01 to 1.03)	<0.001
Current smoker	80 256	33.7	1.19 (1.17 to 1.21)		<0.001	1.07 (1.05 to 1.09)	<0.001
Alcohol status**‡**							
Never drinker	68 031	30.5	1 (ref)		–	1 (ref)	–
Former drinker	66 495	36.6	0.97 (0.94 to 0.99)		0.007	0.93 (0.91 to 0.96)	<0.001
Current drinker	1 173 159	37.7	1.01 (1.00 to 1.03)		0.13	1.09 (1.06 to 1.11)	<0.001
Family history of CVD (ref: No)							
Yes	492 242	45.8	1.35 (1.34 to 1.36)		<0.001	1.30 (1.29 to 1.31)	<0.001
Prior CVD (ref: No)							
Yes	53 546	78.8	3.96 (3.88 to 4.05)		<0.001	3.56 (3.47 to 3.64)	<0.001
Diabetes (ref: No)							
Yes	35 900	64.4	2.29 (2.24 to 2.35)		<0.001	1.56 (1.53 to 1.60)	<0.001
Frequency of falls**‡**							
0	1 104 955	36.2	1 (ref)		–	1 (ref)	–
1	133 698	42.1	1.13 (1.12 to 1.14)		<0.001	1.00 (0.99 to 1.02)	0.57
>2	67 593	44.7	1.36 (1.34 to 1.38)		<0.001	1.01 (0.99 to 1.03)	0.37
Season at the time of enrolment							
Winter	328 563	38.8	1 (ref)		–	1 (ref)	–
Spring	344 289	36.2	0.89 (0.88 to 0.90)		<0.001	0.87 (0.86, 0.88)	<0.001
Summer	315 452	35.4	0.85 (0.84 to 0.86)		<0.001	0.82 (0.81 to 0.83)	<0.001
Autumn	324 247	38.5	0.97 (0.96 to 0.98)		<0.001	0.98 (0.97 to 0.99)	0.001

* A univariate logistic regression model for each covariate (adjusted for age and sex).

† A multivariate logistic regression model including all the covariates in table 1 except ‘Number of blood pressure lowering medications’ due to its high correlation with the outcome/other covariates.

‡ Sex prefer not to answer and missing in age, BMI, education, smoking and drinking status and falls were excluded due to small percentages.

BMI, body mass index; CVD, cardiovascular disease.

**Figure 2:**
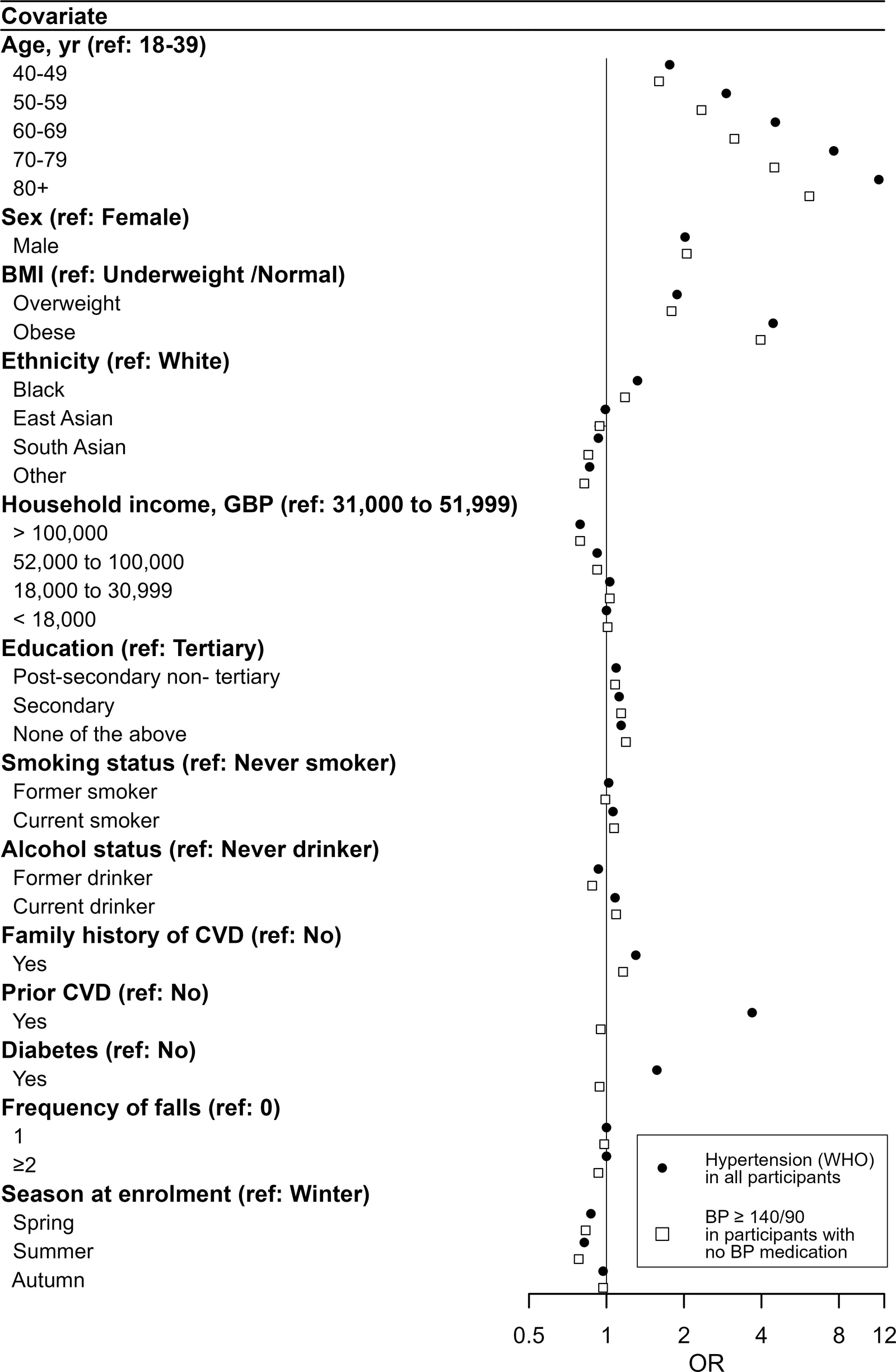
OR of (1) hypertension defined using WHO criteria (solid circle) in all participants, (N=1 312 551) and (2) hypertension defined by BP ≥140/90 mm Hg (open square) in participants with no blood pressure lowering medications (N=1 129 675). BMI, body mass index; BP, blood pressure; CVD, cardiovascular disease.

### Detection of hypertension

Among those with hypertension, detection of the diagnosis generally tracked with the risk factors for hypertension (online supplemental table S2, figure 2). Older age, male sex, black ethnicity, higher body mass index (BMI), family history of CVD, personal history of CVD or diabetes were associated with a higher probability of hypertension detection. South and East Asians who were hypertensive were also more likely to be detected than White British. Conversely, younger, female, white and lean individuals who did not have a family history of CVD or personal history of diabetes were less likely to be detected as having hypertension. A range of other health conditions were associated with higher risks of hypertension in age- and sex-adjusted and multivariable-adjusted analyses (online supplemental table S3) in addition to higher odds of hypertension detection (online supplemental table S4).

### Treatment and control of hypertension

When the definition of hypertension was restricted to those with high BP recorded at enrolment (ie, those on antihypertensive medications were excluded from the analyses), although associations with demographic and lifestyle risk factors were largely unaltered, personal history of CVD and diabetes were unrelated to hypertension in the multivariate analyses (online supplemental table S5). Using a more extreme measure of SBP (≥160 mm Hg) associations were broadly similar, although the associations with age were stronger (online supplemental table S6). Using a less extreme measure of SBP (≥130–139) associations were also comparable, although the associations with age were weaker (online supplemental table S7).

Among those with detected hypertension, use of antihypertensive medications was associated with substantially reduced risk of high BP with a dose-response relationship with the one, two or three medications (online supplemental table S8). There was no apparent relationship between reporting a history of falls and being on antihypertensive medications in the population with detected hypertension (online supplemental table S9) or among participants older than 60 years of age (data not shown).

## Discussion

In this contemporary large survey of BP levels in England, over one-third (37.2%) of participants had hypertension defined using WHO criteria for hypertension, and only half of these had been detected. Among those with no self-report of hypertension diagnosis, 33.2% male and 21.2% female had BP levels ≥140/90 mm Hg. Among those who reported use of one or more antihypertensive medications, one half (50.2%) had BP levels ≥140/90 mm Hg.

The prevalence of hypertension showed that the ‘rule of halves’ still broadly applies with approximately half of hypertension being undetected and half of those treated being under-treated.^11^ The findings on prevalence were similar to previous estimates based on the much smaller ONS household surveys that reported 32% of adult participants had hypertension in 2015–2019 and the percentage of undiagnosed hypertension among adults with hypertension was 29%.^4^ Consistent with results of previous studies, the major risk factors for hypertension included increasing age, male sex, obesity and Black ethnicity, family history of CVD or a personal history of CVD or diabetes. Although there is concern that treatment of hypertension is associated with postural hypotension and a higher risk of falls among the elderly, we observed no such association in data limited to past history of falls.

The most striking finding of this survey was the high absolute number and the high proportion of participants with undiagnosed hypertension; about 60% of those with a BP of ≥140/90 mm Hg had undetected hypertension and were not taking antihypertensive medications. Although some of the demographic risk factors were associated with hypertension, there were no demographic subgroups in which the prevalence of hypertension was not substantial, suggesting that detection and treatment of hypertension requires population-wide approaches in addition to initiation of medication in population subgroups with high absolute risks of CVD. For example, population-wide approaches are required to reduce BP including limiting salt consumption, alcohol consumption and levels of obesity and promoting exercise and intake of fresh fruit and vegetables.

The risk factors for undiagnosed hypertension were mostly the inverse of the risk factors for hypertension with a higher proportion of younger, lean, White ethnicity and individuals with no family history of CVD, personal history of CVD or diabetes being more likely to have undiagnosed hypertension. When those taking antihypertensive medications were excluded from the analyses, the demographic and lifestyle risk factors for measured BP ≥140/90 mm Hg were similar, however, prior CVD and diabetes were no longer associated with uncontrolled hypertension suggesting that primary care is effective in controlling BP among patients with these comorbidities.

Our main analysis excluded participants with serious health conditions because such conditions can influence BP, often require antihypertensive treatment and thereby may distort analysis depending on the propensity for these participants to join a health research study. However, we conducted a supplementary analysis that included these participants. The results were highly consistent with those of the main analysis and the overall interpretations remained unchanged.

The chief strengths of this study included standardised BP measurements in over 1 million participants in a wide range of geographic regions in England recorded between 2022 and 2025, in addition to the clinic measurements of BMI and detailed self-reported questionnaire data. The study also had several limitations, including the non-random sampling (although the prevalence estimates of hypertension were comparable to those obtained by ONS). Data on the Index of Multiple Deprivation were unavailable, although data on individual family income and personal years of education may be better proxies for individual socioeconomic status than a postcode-based Index. The study relied on self-reported data for medications. Information from primary care and other ambulatory-based records was not available so we were not able to validate the estimated hypertension prevalence (based on WHO definition). The absence of data on factors related to clinical touchpoints for hypertension management, including healthcare utilisation and access to primary care or pharmacy services were also limitations as we do not know which participants were effectively engaged with primary care. However, factors such as family history of CVD, prior CVD, diabetes were associated with reduced risk of undetected hypertension suggesting that hypertension management is more effective in patients with conditions that specifically require BP control. Although data from OFH were limited to those collected in England, enrolment for this study has commenced in Scotland, Wales and Northern Ireland and comparable measurements should be available for these regions in the UK in the next few years.

As a survey, OFH is limited to BP measurements recorded at one point in time, which were only approximate estimates of long-term average levels of BP. The National Institute for Health and Care Excellence (NICE) guidelines on diagnosis of hypertension advocate use of ambulatory BP monitoring or home BP monitoring to confirm the diagnosis of hypertension prior to initiating treatment and emphasise desirability of more frequent BP measurements in individuals with type 2 diabetes.^12^ In a NICE review of the concordance of clinic BP measurements ≥140/90 mm Hg compared with ambulatory BP measurements ≥135/85 mm Hg (the recommended threshold for diagnosis) the median sensitivity of the clinic measurement was 81% and the median specificity was 76%.^13^ This suggests that a one-time measure of clinic BP is a good predictor of ‘true’ hypertension but requires confirmation by ambulatory BP measurements or multiple home measurements due to concerns about ‘white coat’ hypertension and regression to the mean.

The hypertension ‘cascade of care’ describes the steps that are required to manage hypertension in the population summarised as detection, treatment and control. Consistent with previous reports from UK Biobank, the findings of this study suggest that detection and treatment of hypertension may be adequate for individuals with prior CVD and diabetes, but there was a very large proportion of participants with undiagnosed and inadequately treated hypertension in the overall population.^14^ Recent evidence from England demonstrates that, despite substantial improvements in hypertension detection and control during the 2000s, progress has plateaued since around 2011, with indications of worsening trends following the COVID-19 pandemic, possibly because disruptions to healthcare delivery may have had lasting effects. Thus, the current hypertension prevalence levels in England are similar to those observed two decades ago.^15^ Analysis of primary care records in the East Midlands Genvasc study indicated that the majority of participants with a Health Check measurement of BP ≥140/90 mm Hg were not prescribed antihypertensive medication, and likewise, about one-third of those classified as being at high risk of CVD by Cardiovascular Risk Score (QRISK2) criteria did not have any record of follow-up in primary care.^16^ In a survey of 27 general practices and health centres across five English National Health Service regions, only 37.8% of patients on BP-lowering medications had BP levels <140/90 mm Hg.^17^ The 15.3% prevalence of hypertension in those less than 40 years of age is a cause for concern since young adults are ineligible for an NHS Health Check. Current guidelines that stress multifactorial CVD risk assessment may also have the consequence of restricting access to antihypertensive treatment, particularly in younger adults at lower 10-year risks of CVD but higher lifetime risks of CVD, despite hypertension being a major cause of stroke at any age.

The findings of this study reinforce evidence that substantial gaps in hypertension detection and management persist and suggest that the current recommendations for detection and treatment of hypertension in England are not fit for purpose and require a population-wide approach to complement the initiation of antihypertensive medication to population subgroups at high 10 years risks of CVD. The current NICE guidelines for management of hypertension require multiple confirmatory steps between the initial BP measurement and a clinical diagnosis, and recommend lifestyle advice before the initiation of treatment. These requirements may contribute to a ‘leaky cascade of care’, with many cases of hypertension remaining undiagnosed and inadequately treated. The workload in primary care focusing on clinical management of established diseases leaves few opportunities for disease prevention.^18^ The international PURE study demonstrated that detection and control are strongly influenced by health system factors, including access to primary care and availability of treatment.^19^ A systematic review of global hypertension studies reported that, from 2000 to 2020, high-income countries experienced increases in awareness (57.7% to 69.2%), treatment (42.9% to 66.3%) and control (16.4% to 40.2%). More modest gains were observed in low- and middle-income countries, where awareness rose from 29.1% to 46.1%, treatment from 20.7% to 30.8% and control from 6.4% to 13.6%.^20^ The Non-Communicable Disease Risk Factor collaboration (NCD-RisC) analyses highlighted marked variation in hypertension detection, treatment and control across countries, with the 2021 global analysis also finding that some middle-income countries now outperform many high-income settings.^21,22^ These findings suggest that, even in comparatively well-resourced healthcare systems such as the UK, there needs to be improvement in hypertension detection and management. Since 2021, pharmacies in the UK have provided facilities for measurement of BP and ambulatory BP measurements prior to referral to a GP and it is possible that extending this approach may improve detection and treatment of hypertension. However, the ‘silent killer’ still currently stalks millions of NHS patients resulting in a substantial associated burden of premature cardiovascular morbidity and mortality in this population.^3^

## Supplementary material

10.1136/bmjph-2025-004815 online supplemental file 1

## Data Availability

Data may be obtained from a third party and are not publicly available.
