# Supplementary material for: Hypertension prevalence, detection, treatment and control among 1.4 million adults in England: a cross-sectional analysis from the Our Future Health study in 2022–2025
Source: BMJ Public Health. 2026 Sep 23;4(3):e004815. doi: 10.1136/bmjph-2025-004815 (PMC13630168; doi:10.1136/bmjph-2025-004815)
Supplement: Supplementary file 1 [file bmjph-4-3-s001.docx]

**Supplementary Appendix**

Table of Contents

[Supplementary Methods	2](#_Toc227663342)

[Variable definitions	2](#_Toc227663343)

[Supplementary Figures and Tables	3](#_Toc227663344)

[Figure S1. Study flowchart.	3](#_Toc227663345)

[Table S1. Percentages (95% CI) of (i) hypertension based on WHO guidelines, (ii) hypertension based on alternative guidelines, (iii) self-reported of hypertension diagnosis, and (iv) no self-reported of hypertension diagnosis in Our Future Health study population^1^.	4](#_Toc227663346)

[Table S2: Associations of risk factors with detection of hypertension (Detected vs. Undetected [reference]) among participants with hypertension^4^ (N=488,729)	5](#_Toc227663347)

[Table S3. Logistic regression models identifying individual types of comorbidities associated with hypertension using WHO criteria (Yes/No) among all study population (N=1,312,551)	8](#_Toc227663348)

[Table S4. Logistic regression models identifying individual types of comorbidities associated with detection (Detected vs Undetected (reference)) among participants with hypertension (N=488,729)	9](#_Toc227663349)

[Table S5: Logistic regression models identifying factors associated with hypertension (>=140/90 vs. <140/90 mmHg (reference)) among participants who did not report blood pressure lowering medication (N= 1,129,675)	10](#_Toc227663350)

[Table S6: Logistic regression models identifying factors associated with hypertension (SBP >=160 vs. <140 mmHg (reference)) among participants who did not report blood pressure lowering medication, excluding those with mean SBP in [140-159] mmHg (N= 933,116)	13](#_Toc227663351)

[Table S7: Logistic regression models identifying factors associated with hypertension (SBP in [130-140] vs <130 mmHg (reference)) among participants who did not report BP lowering medication, excluding those with mean SBP >140 mmHg (N= 899,124)	16](#_Toc227663352)

[Table S8: Logistic regression models identifying factors associated with hypertension (BP >=140/90 vs. <140/90 mm Hg (reference)) among study population with detected hypertension (N= 212,431)	19](#_Toc227663353)

[Table S9: Logistic regression models identifying factors associated with indicator of falls (Yes vs. No (reference)) among study population with detected hypertension (N= 212,431)	22](#_Toc227663354)

[Supplementary analysis including participants with serious health conditions (kidney failure, heart failure, liver failure, and cancer other than basal/squamous cell skin cancer)	25](#_Toc227663355)

[Figure S2. Study flowchart including participants with serious health conditions	25](#_Toc227663356)

[Figure S3. Prevalence of hypertension by age and sex	26](#_Toc227663357)

[Table S10: Associations of risk factors with hypertension defined using WHO criteria (Yes vs. No [reference]) in the study population (N=1,428,082)	27](#_Toc227663358)

# Supplementary Methods

## Variable definitions

Taking medications for hypertension was defined as “Treated” if “2 Blood pressure medication” was answered for the question “*Do you regularly take any of the following medications?*” in Questionnaire V1 or if “7 Heart or circulatory health (e.g. high blood pressure or stroke)” was answered for the question “*Do you regularly take medications for any of the following reasons?*”, with any of the medications “4 Calcium channel blocker (e.g. verapamil, diltiazem); 6 Blood pressure medication (e.g. enalapril, lisinopril, perindopril, ramipril, candesartan, irbesartan, losartan, valsartan and Olmesartan); 7 Beta blocker (e.g. bisoprolol, atenolol); 8 Diuretic (e.g. Furosemide, Fendroflumethiazide, Amiloride, Bumetanide, Metalozone, Spironolactone)” selected for the follow-up question “*Do you regularly take any of the following medication for heart or circulatory disorders?*” in Questionnaire V2. Otherwise, medications for hypertension = “Untreated”.

Detection of hypertension was defined as receiving medications for hypertension or report of a prior diagnosis of hypertension by a health professional, where the latter was a Yes if “4 High blood pressure” was answered for the baseline question “*Has a doctor ever told you that you have had any of the following condition?*” in Questionnaire V1 or if “9 Heart or circulatory disease (e.g. high blood pressure or stroke)” was answered for the question “*Have you ever been diagnosed with any of the following by a doctor or other health professional?*”, with “9 High Blood Pressure (Hypertension) [Not hypertension during pregnancy.]” selected for the follow-up question “*Have you ever been diagnosed with any of the following heart or circulatory diseases by a doctor or other health professional?*” in Questionnaire V2. Otherwise, Detection = “Undetected”.

# Supplementary Figures and Tables

## Figure S1. Study flowchart.

Figure S1. Study flowchart. Serious health conditions include kidney failure, heart failure, liver failure, and cancer other than basal/squamous cell skin cancer. “**Hypertension**” was defined based on the WHO guidelines as the number of participants who (i) had mean SBP of ≥140 mmHg or mean DBP ≥ 90 mmHg, or (ii) were receiving antihypertensive medications. “**Detected**” was defined as report of a prior diagnosis of hypertension by a health professional or receiving antihypertensive medication, among those who were hypertensive. “**Treated**” was defined as report of hypertensive medication. “**Controlled**” was defined if SBP was <140 mmHg and DBP <90 mmHg among those taking antihypertensive medication. OFH: Our Future Health; BP: blood pressure; SBP: systolic blood pressure; DBP: diastolic blood pressure; WHO: World Health Organization.

## Table S1. Percentages (95% CI) of (i) hypertension based on WHO guidelines, (ii) hypertension based on alternative guidelines, (iii) self-reported of hypertension diagnosis, and (iv) no self-reported of hypertension diagnosis in Our Future Health study population^1^.

|  | **Crude prevalence, %^2^ (95% CI)** | | **Age- and sex-standardised prevalence^5^, %^2^ (95% CI)** | |
| --- | --- | --- | --- | --- |
|  | Male (N=579,611) | Female (N=732,174) | Male (N=579,611) | Female (N=732,174) |
| **(i) Hypertension** **(WHO guidelines)**^3^ | 47.26 (47.13, 47.38) | 29.31 (29.20, 29.41) | 41.89 (41.73, 42.05) | 28.60 (28.47, 28.72) |
| SBP <120 and DBP <80, mmHg | 3.80 (3.72, 3.87) | 4.61 (4.53, 4.70) | 2.58 (2.53, 2.63) | 4.16 (4.08, 4.24) |
| SBP 120-129 and DBP <80 | 3.61 (3.54, 3.68) | 3.63 (3.55, 3.71) | 2.38 (2.34, 2.43) | 2.48 (2.42, 2.53) |
| SBP 130-139 or DBP 80-89 | 10.76 (10.64, 10.87) | 11.01 (10.88, 11.15) | 7.34 (7.25, 7.42) | 8.42 (8.31, 8.52) |
| SBP ≥140 or DBP ≥90 | 81.83 (81.69, 81.98) | 80.74 (80.58, 80.91) | 87.70 (97.34, 88.07) | 84.94 (84.54, 85.34) |
| **(ii) Hypertension** **(alternative guideline)**^4^ | 44.42 (44.29, 44.55) | 28.23 (28.13, 28.33) | 39.65 (39.50, 39.81) | 27.43 (27.31, 27.54) |
| SBP <120 and DBP <80, mmHg | 2.14 (2.08, 2.19) | 3.13 (3.06, 3.21) | 1.53 (1.49, 1.57) | 2.52 (2.46, 2.58) |
| SBP 120-129 and DBP <80 | 2.40 (2.34, 2.46) | 2.95 (2.88, 3.03) | 1.69 (1.64, 1.73) | 2.04 (1.99, 2.09) |
| SBP 130-139 or DBP 80-89 | 8.41 (8.30, 8.52) | 10.10 (9.97, 10.23) | 6.25 (6.16, 6.33) | 8.12 (8.01, 8.23) |
| SBP ≥140 or DBP ≥90 | 87.06 (86.93, 87.19) | 83.82 (83.66, 83.98) | 90.53 (90.16, 90.91) | 87.32 (86.91, 87.74) |
| **(iii) Self-report of hypertension diagnosis** | 13.74 (13.65, 13.83) | 9.50 (9.43, 9.57) | 11.06 (10.99, 11.14) | 9.31 (9.24, 9.38) |
| SBP <120 and DBP <80, mmHg | 6.91 (6.73, 7.09) | 9.30 (9.09, 9.52) | 6.01 (5.85, 6.17) | 9.66 (9.42, 9.90) |
| SBP 120-129 and DBP <80 | 7.75 (7.56, 7.94) | 8.78 (8.57, 8.99) | 6.53 (6.37, 6.70) | 6.38 (6.22, 6.54) |
| SBP 130-139 or DBP 80-89 | 27.19 (26.88, 27.50) | 30.01 (29.67, 30.35) | 27.56 (27.20, 27.93) | 30.96 (30.54, 31.38) |
| SBP ≥140 or DBP ≥90 | 58.16 (57.81, 58.50) | 51.91 (51.54, 52.28) | 59.89 (59.35, 60.44) | 53.00 (52.45, 53.54) |
| **(iv) No self-report of hypertension diagnosis** | | | | |
| SBP <120 and DBP <80, mmHg | 17.05 (16.95, 17.15) | 39.98 (39.87, 40.10) | 18.12 (17.99, 18.24) | 40.16 (40.01, 40.31) |
| SBP 120-129 and DBP <80 | 13.69 (13.60, 13.79) | 10.62 (10.54, 10.69) | 14.85 (14.74, 14.96) | 10.56 (10.48, 10.64) |
| SBP 130-139 or DBP 80-89 | 33.69 (33.56, 33.82) | 28.70 (28.59, 28.81) | 33.87 (33.71, 34.03) | 28.08 (27.95, 28.20) |
| SBP ≥140 or DBP ≥90 | 35.57 (35.44, 35.70) | 20.70 (20.60, 20.80) | 33.17 (33.01, 33.32) | 21.20 (21.09, 21.31) |

^1^: Study population which excluded serious health conditions, missing/implausible blood pressure measurements. Here, intersex/other was also excluded due to small sample size.

^2^: The %’s for “Hypertension (WHO guidelines)”, “Hypertension (alternative guideline)”, “Self-report of hypertension diagnosis” and “No self-report of hypertension diagnosis” are based on the total population (N=579,611 for men and 732,174 for women). The %’s of each blood pressure level were based on 273,902 men and 214,571 women, respectively, for “Hypertension (WHO guidelines)”, 257,463 men and 206,700 women for “Hypertension (alternative guideline)”, 79,628 men and 69,556 women for “Self-report of hypertension diagnosis”, and 499,983 men and 662,618 women for “No self-report of hypertension diagnosis”.

^3^: (WHO guidelines) High blood pressure is defined as either receiving blood pressure lowering medication or elevated blood pressure (SBP≥140 or DBP≥90 mm Hg).

^4^: (Alternative guideline) High blood pressure is defined as either self-reported diagnosed hypertension or elevated blood pressure (SBP≥140 or DBP≥90 mm Hg).

^5^: Using the age and sex structure of the [ONS population estimates for England at mid-2023](https://www.ons.gov.uk/peoplepopulationandcommunity/populationandmigration/populationestimates/datasets/populationestimatesforukenglandandwalesscotlandandnorthernireland) for direct age-standardisation.

## Table S2: Associations of risk factors with detection of hypertension (Detected vs. Undetected [reference]) among participants with hypertension^4^ (N=488,729)

|  |  |  | **Age and sex adjusted^1^** | | | **Multivariable adjusted^2^** | |
| --- | --- | --- | --- | --- | --- | --- | --- |
| **Covariate** | **N** | **Detected, %** | **OR (95%CI)** | | **P value** | **OR (95%CI)** | **P value** |
| Age, year |  |  |  | |  |  |  |
| 18-39 | 47656 | 11.8 | 1 (ref) | | - | 1 (ref) | - |
| 40-49 | 53864 | 24.4 | 2.42 (2.34, 2.51) | | <0.001 | 2.19 (2.11, 2.27) | <0.001 |
| 50-59 | 108633 | 36.0 | 4.23 (4.10, 4.36) | | <0.001 | 3.69 (3.57, 3.80) | <0.001 |
| 60-69 | 153913 | 46.8 | 6.60 (6.41, 6.80) | | <0.001 | 5.56 (5.38, 5.73) | <0.001 |
| 70-79 | 106269 | 56.3 | 9.69 (9.40, 9.98) | | <0.001 | 8.17 (7.91, 8.44) | <0.001 |
| 80+ | 18283 | 61.5 | 11.97 (11.49, 12.47) | | <0.001 | 9.88 (9.46, 10.33) | <0.001 |
| Sex^3^ |  |  |  |  |  |  |  |
| Female | 214571 | 40.5 | 1 (ref) | | - | 1 (ref) | - |
| Male | 273902 | 41.7 | 1.09 (1.07, 1.10) | | <0.001 | 1.02 (1.01, 1.03) | 0.003 |
| BMI |  |  |  | |  |  |  |
| Underweight /Normal | 110712 | 36.6 | 1 (ref) | | - | 1 (ref) | - |
| Overweight | 197522 | 40.9 | 1.26 (1.24, 1.28) | | <0.001 | 1.26 (1.23, 1.28) | <0.001 |
| Obese | 179535 | 44.2 | 1.79 (1.76, 1.82) | | <0.001 | 1.70 (1.67, 1.73) | <0.001 |
| Ethnicity |  |  |  |  |  |  |  |
| White | 450322 | 41.4 | 1(ref) | | - | 1(ref) | - |
| Black | 8204 | 41.1 | 1.56 (1.49, 1.64) | | <0.001 | 1.58 (1.50, 1.66) | <0.001 |
| South Asian | 12739 | 38.8 | 1.38 (1.32, 1.43) | | <0.001 | 1.34 (1.28, 1.40) | <0.001 |
| East Asian | 3556 | 34.7 | 1.02 (0.95, 1.10) | | 0.57 | 1.32 (1.22, 1.42) | <0.001 |
| Other | 13908 | 35.9 | 1.18 (1.13, 1.22) | | <0.001 | 1.17 (1.13, 1.22) | <0.001 |
| Household income, GBP |  |  |  |  |  |  |  |
| ≥ 100,000 | 45167 | 32.9 | 0.90 (0.88, 0.92) | | <0.001 | 0.99 (0.96, 1.01) | 0.35 |
| 52,000 to 99,999 | 111621 | 35.5 | 0.93 (0.91, 0.94) | | <0.001 | 0.96 (0.94, 0.98) | <0.001 |
| 31,000 to 51,999 | 108474 | 42.5 | 1 (ref) | | - | 1 (ref) | - |
| 18,000 to 30,999 | 88030 | 46.7 | 1.07 (1.05, 1.09) | | <0.001 | 1.02 (1.00, 1.04) | 0.05 |
| < 18,000 | 51672 | 47.6 | 1.13 (1.11, 1.16) | | <0.001 | 1.01 (0.99, 1.04) | 0.27 |
| Prefer not to  answer/Missing | 83765 | 41.7 | 0.91 (0.90, 0.93) | | <0.001 | 0.93 (0.91, 0.95) | <0.001 |
| Education |  |  |  |  |  |  |  |
| Tertiary | 288084 | 39.6 | 1 (ref) | | - | 1 (ref) | - |
| Post-secondary  non- tertiary | 37480 | 47.8 | 1.05 (1.02, 1.07) | | <0.001 | 1.02 (1.00, 1.05) | 0.06 |
| Secondary | 122550 | 41.3 | 0.96 (0.94, 0.97) | | <0.001 | 0.94 (0.93, 0.95) | <0.001 |
| None of the above | 32751 | 47.9 | 0.95 (0.93, 0.98) | | <0.001 | 0.87 (0.85, 0.89) | <0.001 |
| Prefer not to  answer/Missing | 7864 | 34.8 | 0.78 (0.74, 0.82) | | <0.001 | 0.80 (0.76, 0.85) | <0.001 |
| Smoking status |  |  |  |  |  |  |  |
| Never smoker | 291183 | 39.4 | 1 (ref) | | - | 1 (ref) | - |
| Former smoker | 158776 | 46.0 | 1.16 (1.15, 1.18) | | <0.001 | 1.10 (1.08, 1.11) | <0.001 |
| Current smoker | 27035 | 33.9 | 1.07 (1.04, 1.10) | | <0.001 | 1.01 (0.98, 1.04) | 0.45 |
| Prefer not to  answer/Missing | 11735 | 35.9 | 0.95 (0.91, 0.99) | | 0.01 | 0.94 (0.90, 0.98) | 0.008 |
| Alcohol status |  |  |  |  |  |  |  |
| Never drinker | 20782 | 39.7 | 1 (ref) | | - | 1 (ref) | - |
| Former drinker | 24331 | 48.1 | 1.18 (1.13, 1.22) | | <0.001 | 1.19 (1.14, 1.24) | <0.001 |
| Current drinker | 441912 | 40.9 | 0.86 (0.83, 0.88) | | <0.001 | 0.99 (0.96, 1.03) | 0.63 |
| Prefer not to  answer/Missing | 1704 | 28.5 | 0.57 (0.51, 0.64) | | <0.001 | 0.82 (0.73, 0.93) | 0.002 |
| Family history of CVD |  |  |  |  |  |  |  |
| No | 173699 | 34.1 | 1 (ref) | | - | 1 (ref) | - |
| Yes | 315030 | 49.4 | 1.60 (1.58, 1.62) | | <0.001 | 1.50 (1.48, 1.52) | <0.001 |
| Prior CVD |  |  |  |  |  |  |  |
| No | 446542 | 36.7 | 1 (ref) | | - | 1 (ref) | - |
| Yes | 42187 | 88.1 | 10.61 (10.29, 10.95) | | <0.001 | 9.88 (9.57, 10.19) | <0.001 |
| Diabetes |  |  |  |  |  |  |  |
| No | 465625 | 39.6 | 1 (ref) | | - | 1 (ref) | - |
| Yes | 23104 | 71.9 | 3.65 (3.54, 3.76) | | <0.001 | 3.03 (2.94, 3.13) | <0.001 |
| Frequency of falls |  |  |  |  |  |  |  |
| 0 | 400197 | 39.5 | 1 (ref) | | - | 1 (ref) | - |
| 1 | 56277 | 47.2 | 1.21 (1.19, 1.23) | | <0.001 | 1.12 (1.10, 1.14) | <0.001 |
| ≥2 | 30232 | 53.1 | 1.68 (1.64, 1.72) | | <0.001 | 1.33 (1.30, 1.37) | <0.001 |
| Prefer not to  answer/Missing | 2023 | 28.6 | 0.76 (0.68, 0.84) | | <0.001 | 0.79 (0.70, 0.88) | 0.001 |
| Season at the time of enrolment | | |  |  |  |  |  |
| Winter | 127526 | 39.4 | 1 (ref) | | - | 1 (ref) | - |
| Spring | 124721 | 42.3 | 1.14 (1.12, 1.16) | | <0.001 | 1.14 (1.12, 1.16) | <0.001 |
| Summer | 111660 | 42.6 | 1.17 (1.15, 1.19) | | <0.001 | 1.16 (1.14, 1.18) | <0.001 |
| Autumn | 124822 | 40.5 | 1.05 (1.03, 1.07) | | <0.001 | 1.05 (1.03, 1.07) | <0.001 |

**^1^**: A univariate logistic regression model for each covariate (adjusted for age and sex).

**^2^**: A multivariate logistic regression model including all the covariates in Table 1 except ‘Number of blood pressure lowering medications’ due to its high correlation with the outcome/other covariate.

**^3^**: “Other/Intersex” and “Prefer not to answer” were omitted here due to small percentages (<0.1%)

^4^: Defined based on the WHO guidelines as participants who (i) had mean SBP of ≥140 mmHg or mean DBP ≥ 90 mmHg, or (ii) were receiving antihypertensive medications.

## Table S3. Logistic regression models identifying individual types of comorbidities associated with hypertension using WHO criteria (Yes/No) among all study population (N=1,312,551)

|  |  |  |  | **Age and sex adjusted^1^** | | **Multivariable adjusted^2^** | |
| --- | --- | --- | --- | --- | --- | --- | --- |
| **Covariate** | **Level** | **N** | **Hypertension, %** | **OR (95%CI)** | **P value** | **OR (95%CI)** | **P value** |
| CVD | No | 1259005 | 35.5 | 1 | - | 1 | - |
|  | Yes | 53546 | 78.8 | 3.96 (3.88, 4.05) | <0.001 | 3.56 (3.47, 3.64) | <0.001 |
| Arrhythmia | No | 1281135 | 36.2 | 1 | - | 1 | - |
|  | Yes | 31416 | 77.6 | 3.79 (3.69, 3.90) | <0.001 | 2.88 (2.79, 2.96) | <0.001 |
| Diabetes | No | 1276651 | 36.5 | 1 | - | 1 | - |
|  | Yes | 35900 | 64.4 | 2.29 (2.24, 2.35) | <0.001 | 1.56 (1.53, 1.60) | <0.001 |
| Asthma or COPD | No | 1207068 | 36.9 | 1 | - | 1 | - |
|  | Yes | 105483 | 40.8 | 1.21 (1.19, 1.22) | <0.001 | 1.06 (1.05, 1.08) | <0.001 |
| Migraines | No | 1299625 | 37.2 | 1 | - | 1 | - |
|  | Yes | 12926 | 36.0 | 1.17 (1.13, 1.22) | <0.001 | 1.00 (0.96, 1.04) | 0.86 |
| Epilepsy | No | 1302724 | 37.2 | 1 | - | 1 | - |
|  | Yes | 9827 | 36.3 | 1.03 (0.98, 1.07) | 0.27 | 0.90 (0.86, 0.94) | <0.001 |
| Anxiety | No | 1154683 | 38.0 | 1 | - | 1 | - |
|  | Yes | 157868 | 31.9 | 1.23 (1.22, 1.25) | <0.001 | 0.99 (0.98, 1.01) | 0.29 |
| Depression | No | 1119781 | 37.7 | 1 | - | 1 | - |
|  | Yes | 192770 | 34.4 | 1.22 (1.20, 1.23) | <0.001 | 0.96 (0.95, 0.97) | <0.001 |
| Osteoarthritis | No | 1227541 | 36.2 | 1 | - | 1 | - |
|  | Yes | 85010 | 51.9 | 1.26 (1.24, 1.28) | <0.001 | 1.03 (1.01, 1.04) | <0.001 |
| Other joint disorder | No | 1300012 | 37.0 | 1 | - | 1 | - |
|  | Yes | 12539 | 65.9 | 1.80 (1.73, 1.87) | <0.001 | 1.30 (1.25, 1.35) | <0.001 |

**^1^**: A univariate logistic regression model for each covariate (adjusted for age and sex).

**^2^**: A multivariate logistic regression model including all the covariates in Table 1 except ‘Number of blood pressure lowering medications’ due to its high correlation with the outcome/other covariate.

## Table S4. Logistic regression models identifying individual types of comorbidities associated with detection (Detected vs Undetected (reference)) among participants with hypertension (N=488,729)

|  |  |  |  | **Age and sex adjusted^1^** | | **Multivariable adjusted^2^** | |
| --- | --- | --- | --- | --- | --- | --- | --- |
| **Covariate** | **Level** | **N** | **Detected, %** | **OR (95%CI)** | **P value** | **OR (95%CI)** | **P value** |
| CVD | No | 446542 | 36.7 | 1 | - | 1 | - |
|  | Yes | 42187 | 88.1 | 10.61 (10.29, 10.95) | <0.001 | 9.88 (9.57, 10.19) | <0.001 |
| Arrhythmia | No | 464343 | 38.6 | 1 | - | 1 | - |
|  | Yes | 24386 | 90.4 | 12.54 (12.00, 13.11) | <0.001 | 10.10 (9.65, 10.56) | <0.001 |
| Diabetes | No | 465625 | 39.6 | 1 | - | 1 | - |
|  | Yes | 23104 | 71.9 | 3.65 (3.54, 3.76) | <0.001 | 3.03 (2.94, 3.13) | <0.001 |
| Asthma or COPD | No | 445686 | 40.5 | 1 | - | 1 | - |
|  | Yes | 43043 | 47.4 | 1.37 (1.34, 1.40) | <0.001 | 1.22 (1.19, 1.25) | <0.001 |
| Migraines | No | 484072 | 41.1 | 1 | - | 1 | - |
|  | Yes | 4657 | 50.2 | 1.72 (1.62, 1.83) | <0.001 | 1.36 (1.27, 1.45) | <0.001 |
| Epilepsy | No | 485161 | 41.1 | 1 | - | 1 | - |
|  | Yes | 3568 | 43.1 | 1.20 (1.12, 1.29) | <0.001 | 1.01 (0.94, 1.09) | 0.78 |
| Anxiety | No | 438301 | 40.9 | 1 | - | 1 | - |
|  | Yes | 50428 | 43.5 | 1.66 (1.63, 1.70) | <0.001 | 1.42 (1.39, 1.45) | <0.001 |
| Depression | No | 422363 | 40.5 | 1 | - | 1 | - |
|  | Yes | 66366 | 45.2 | 1.64 (1.61, 1.67) | <0.001 | 1.39 (1.37, 1.42) | <0.001 |
| Osteoarthritis | No | 444628 | 39.9 | 1 | - | 1 | - |
|  | Yes | 44101 | 53.6 | 1.35 (1.32, 1.38) | <0.001 | 1.19 (1.17, 1.22) | <0.001 |
| Other joint disorder | No | 480460 | 40.9 | 1 | - | 1 | - |
|  | Yes | 8269 | 58.3 | 1.71 (1.63, 1.79) | <0.001 | 1.48 (1.41, 1.55) | <0.001 |

**^1^**: A univariate logistic regression model for each covariate (adjusted for age and sex).

**^2^**: A multivariate logistic regression model including all the covariates in Table 1 except ‘Number of blood pressure lowering medications’ due to its high correlation with the outcome/other covariate.

## Table S5: Logistic regression models identifying factors associated with hypertension (>=140/90 vs. <140/90 mmHg (reference)) among participants who did not report blood pressure lowering medication (N= 1,129,675)

|  |  |  | **Age and sex adjusted^1^** | | | **Multivariable adjusted^2^** | |
| --- | --- | --- | --- | --- | --- | --- | --- |
| **Covariate** | **N** | **Hypertension, %** | **OR (95%CI)** | | **P value** | **OR (95%CI)** | **P value** |
| Age, year |  |  |  | |  |  |  |
| 18-39 | 307718 | 14.1 | 1 (ref) | | - | 1 (ref) | - |
| 40-49 | 200049 | 21.5 | 1.66 (1.64, 1.69) | | <0.001 | 1.60 (1.57, 1.62) | <0.001 |
| 50-59 | 250019 | 29.6 | 2.58 (2.55, 2.62) | | <0.001 | 2.34 (2.31, 2.37) | <0.001 |
| 60-69 | 242308 | 36.1 | 3.44 (3.39, 3.49) | | <0.001 | 3.15 (3.10, 3.20) | <0.001 |
| 70-79 | 114343 | 43.8 | 4.69 (4.61, 4.76) | | <0.001 | 4.55 (4.47, 4.63) | <0.001 |
| 80+ | 15142 | 50.8 | 6.00 (5.80, 6.21) | | <0.001 | 6.29 (6.07, 6.52) | <0.001 |
| Sex |  |  |  |  |  |  |  |
| Female | 653716 | 20.8 | 1 (ref) | | - | 1 (ref) | - |
| Male | 475268 | 35.7 | 2.10 (2.08, 2.11) | | <0.001 | 2.11 (2.09, 2.12) | <0.001 |
|  |  |  |  | |  |  |  |
| BMI |  |  |  | |  |  |  |
| Underweight /Normal | 454701 | 16.3 | 1 (ref) | | - | 1 (ref) | - |
| Overweight | 423669 | 29.2 | 1.83 (1.81, 1.85) | | <0.001 | 1.82 (1.80, 1.83) | <0.001 |
| Obese | 249251 | 43.2 | 4.17 (4.12, 4.22) | | <0.001 | 4.08 (4.03, 4.13) | <0.001 |
| Ethnicity |  |  |  |  |  |  |  |
| White | 1003049 | 27.9 | 1(ref) | | - | 1(ref) | - |
| Black | 18477 | 28.7 | 1.37 (1.32, 1.41) | | <0.001 | 1.17 (1.13, 1.22) | <0.001 |
| South Asian | 42470 | 19.4 | 0.79 (0.77, 0.81) | | <0.001 | 0.85 (0.82, 0.87) | <0.001 |
| East Asian | 14785 | 16.9 | 0.66 (0.63, 0.69) | | <0.001 | 0.96 (0.91, 1.00) | 0.06 |
| Other | 50893 | 18.7 | 0.80 (0.78, 0.82) | | <0.001 | 0.82 (0.80, 0.84) | <0.001 |
| Household income, GBP |  |  |  |  |  |  |  |
| ≥ 100,000 | 158094 | 20.3 | 0.70 (0.69, 0.71) | | <0.001 | 0.79 (0.77, 0.80) | <0.001 |
| 52,000 to 99,999 | 312810 | 24.3 | 0.90 (0.88, 0.91) | | <0.001 | 0.92 (0.91, 0.94) | <0.001 |
| 31,000 to 51,999 | 227961 | 29.1 | 1 (ref) | | - | 1 (ref) | - |
| 18,000 to 30,999 | 158191 | 31.8 | 1.06 (1.05, 1.08) | | <0.001 | 1.03 (1.01, 1.04) | <0.001 |
| < 18,000 | 94360 | 31.1 | 1.08 (1.06, 1.10) | | <0.001 | 1.01 (0.99, 1.03) | 0.39 |
| Prefer not to  answer/Missing | 178259 | 29.1 | 1.02 (1.00, 1.03) | | 0.01 | 1.01 (1.00, 1.03) | 0.07 |
| Education |  |  |  |  |  |  |  |
| Tertiary | 756999 | 24.4 | 1 (ref) | | - | 1 (ref) | - |
| Post-secondary  non- tertiary | 63389 | 33.0 | 1.15 (1.13, 1.17) | | <0.001 | 1.08 (1.06, 1.10) | <0.001 |
| Secondary | 246595 | 31.0 | 1.25 (1.23, 1.26) | | <0.001 | 1.13 (1.12, 1.15) | <0.001 |
| None of the above | 45767 | 40.0 | 1.42 (1.39, 1.45) | | <0.001 | 1.19 (1.17, 1.22) | <0.001 |
| Prefer not to  answer/Missing | 16925 | 32.0 | 1.35 (1.30, 1.39) | | <0.001 | 1.19 (1.14, 1.23) | <0.001 |
| Smoking status |  |  |  |  |  |  |  |
| Never smoker | 731833 | 25.6 | 1 (ref) | | - | 1 (ref) | - |
| Former smoker | 296271 | 31.0 | 1.10 (1.09, 1.12) | | <0.001 | 1.00 (0.99, 1.01) | 0.43 |
| Current smoker | 72193 | 26.3 | 1.18 (1.16, 1.20) | | <0.001 | 1.07 (1.05, 1.09) | <0.001 |
| Prefer not to  answer/Missing | 29378 | 26.8 | 1.14 (1.11, 1.17) | | <0.001 | 1.02 (0.99, 1.05) | 0.28 |
| Alcohol status |  |  |  |  |  |  |  |
| Never drinker | 60605 | 22.0 | 1 (ref) | | - | 1 (ref) | - |
| Former drinker | 55885 | 24.6 | 0.91 (0.89, 0.94) | | 0.007 | 0.89 (0.86, 0.91) | <0.001 |
| Current drinker | 1008753 | 27.5 | 1.07 (1.05, 1.09) | | 0.13 | 1.09 (1.07, 1.12) | <0.001 |
| Prefer not to  answer/Missing | 4432 | 28.7 | 1.19 (1.11, 1.28) | | 0.36 | 1.06 (0.98, 1.15) | 0.14 |
| Family history of CVD |  |  |  |  |  |  |  |
| No | 739264 | 24.7 | 1 (ref) | | - | 1 (ref) | - |
| Yes | 390411 | 31.6 | 1.16 (1.15, 1.17) | | <0.001 | 1.16 (1.15, 1.17) | <0.001 |
| Prior CVD |  |  |  |  |  |  |  |
| No | 1111884 | 26.9 | 1 (ref) | | - | 1 (ref) | - |
| Yes | 17791 | 36.2 | 1.04 (1.01, 1.08) | | 0.01 | 0.96 (0.93, 0.99) | 0.01 |
| Diabetes |  |  |  |  |  |  |  |
| No | 1109225 | 26.9 | 1 (ref) | | - | 1 (ref) | - |
| Yes | 20450 | 37.4 | 1.24 (1.21, 1.28) | | <0.001 | 0.95 (0.92, 0.98) | 0.001 |
| Frequency of falls |  |  |  |  |  |  |  |
| 0 | 961304 | 26.7 | 1 (ref) | | - | 1 (ref) | - |
| 1 | 109477 | 29.3 | 1.07 (1.06, 1.09) | | <0.001 | 0.98 (0.97, 0.99) | 0.008 |
| ≥2 | 53087 | 29.6 | 1.15 (1.13, 1.17) | | <0.001 | 0.93 (0.91, 0.95) | <0.001 |
| Prefer not to  answer/Missing | 5807 | 26.3 | 1.15 (1.08, 1.22) | | <0.001 | 0.97 (0.91, 1.04) | 0.39 |
| Season at the time of enrolment | | |  |  |  |  |  |
| Winter | 282997 | 29.0 | 1 (ref) | | - | 1 (ref) | - |
| Spring | 295954 | 25.8 | 0.85 (0.84, 0.86) | | <0.001 | 0.83 (0.82, 0.85) | <0.001 |
| Summer | 271822 | 25.0 | 0.81 (0.80, 0.82) | | <0.001 | 0.79 (0.78, 0.80) | <0.001 |
| Autumn | 278902 | 28.5 | 0.96 (0.95, 0.97) | | <0.001 | 0.97 (0.96, 0.99) | <0.001 |

**^1^**: A univariate logistic regression model for each covariate (adjusted for age and sex).

**^2^**: A multivariate logistic regression model including all the covariates in Table 1 except ‘Number of blood pressure lowering medications’ due to its high correlation with the outcome/other covariate.

**^3^**: “Other/Intersex” and “Prefer not to answer” were omitted here due to small percentages (<0.1%)

## Table S6: Logistic regression models identifying factors associated with hypertension (SBP >=160 vs. <140 mmHg (reference)) among participants who did not report blood pressure lowering medication, excluding those with mean SBP in [140-159] mmHg (N= 933,116)

|  |  |  | **Age and sex adjusted^1^** | | | **Multivariable adjusted^2^** | |
| --- | --- | --- | --- | --- | --- | --- | --- |
| **Covariate** | **N** | **Hypertension, %** | **OR (95%CI)** | | **P value** | **OR (95%CI)** | **P value** |
| Age, year |  |  |  | |  |  |  |
| 18-39 | 283786 | 0.7 | 1 (ref) | | - | 1 (ref) | - |
| 40-49 | 176700 | 1.9 | 2.92 (2.76, 3.09) | | <0.001 | 2.81 (2.65, 2.97) | <0.001 |
| 50-59 | 204263 | 4.5 | 7.20 (6.85, 7.57) | | <0.001 | 6.43 (6.11, 6.77) | <0.001 |
| 60-69 | 180938 | 8.4 | 13.61 (12.97, 14.29) | | <0.001 | 12.11 (11.51, 12.73) | <0.001 |
| 70-79 | 77736 | 14.1 | 23.88 (22.71, 25.10) | | <0.001 | 22.26 (21.12, 23.46) | <0.001 |
| 80+ | 9632 | 20.8 | 36.66 (34.27, 39.21) | | <0.001 | 37.12 (34.58, 39.84) | <0.001 |
| Sex |  |  |  |  |  |  |  |
| Female | 571701 | 3.2 | 1 (ref) | | - | 1 (ref) | - |
| Male | 360828 | 6.7 | 2.00 (1.96, 2.04) | | <0.001 | 2.00 (1.96, 2.04) | <0.001 |
|  |  |  |  | |  |  |  |
| BMI |  |  |  | |  |  |  |
| Underweight /Normal | 404368 | 2.4 | 1 (ref) | | - | 1 (ref) | - |
| Overweight | 341593 | 5.0 | 1.78 (1.73, 1.83) | | <0.001 | 1.75 (1.71, 1.80) | <0.001 |
| Obese | 185422 | 8.6 | 4.24 (4.13, 4.36) | | <0.001 | 4.15 (4.04, 4.27) | <0.001 |
| Ethnicity |  |  |  |  |  |  |  |
| White | 821105 | 4.9 | 1(ref) | | - | 1(ref) | - |
| Black | 15205 | 4.9 | 1.81 (1.68, 1.96) | | <0.001 | 1.51 (1.40, 1.64) | <0.001 |
| South Asian | 38002 | 2.0 | 0.71 (0.66, 0.77) | | <0.001 | 0.75 (0.70, 0.81) | <0.001 |
| East Asian | 13386 | 1.8 | 0.57 (0.50, 0.65) | | <0.001 | 0.82 (0.72, 0.94) | 0.003 |
| Other | 45417 | 2.0 | 0.75 (0.70, 0.80) | | <0.001 | 0.76 (0.71, 0.81) | <0.001 |
| Household income, GBP |  |  |  |  |  |  |  |
| ≥ 100,000 | 137950 | 2.3 | 0.65 (0.62, 0.68) | | <0.001 | 0.72 (0.69, 0.75) | <0.001 |
| 52,000 to 99,999 | 264788 | 3.4 | 0.89 (0.86, 0.91) | | <0.001 | 0.92 (0.89, 0.94) | <0.001 |
| 31,000 to 51,999 | 184901 | 5.1 | 1 (ref) | | - | 1 (ref) | - |
| 18,000 to 30,999 | 125243 | 6.6 | 1.11 (1.07, 1.14) | | <0.001 | 1.06 (1.02, 1.09) | 0.001 |
| < 18,000 | 75784 | 6.2 | 1.11 (1.07, 1.15) | | <0.001 | 1.01 (0.97, 1.05) | 0.558 |
| Prefer not to  answer/Missing | 144450 | 5.7 | 1.11 (1.07, 1.14) | | <0.001 | 1.06 (1.02, 1.09) | 0.001 |
| Education |  |  |  |  |  |  |  |
| Tertiary | 640381 | 3.7 | 1 (ref) | | - | 1 (ref) | - |
| Post-secondary  non- tertiary | 49219 | 7.0 | 1.19 (1.15, 1.24) | | <0.001 | 1.10 (1.06, 1.15) | <0.001 |
| Secondary | 196641 | 5.8 | 1.31 (1.28, 1.34) | | <0.001 | 1.18 (1.16, 1.21) | <0.001 |
| None of the above | 33437 | 10.8 | 1.60 (1.54, 1.67) | | <0.001 | 1.35 (1.29, 1.40) | <0.001 |
| Prefer not to  answer/Missing | 13438 | 5.9 | 1.44 (1.34, 1.56) | | <0.001 | 1.22 (1.12, 1.32) | <0.001 |
| Smoking status |  |  |  |  |  |  |  |
| Never smoker | 611292 | 4.2 | 1 (ref) | | - | 1 (ref) | - |
| Former smoker | 236188 | 5.8 | 1.07 (1.05, 1.09) | | <0.001 | 0.97 (0.94, 0.99) | 0.002 |
| Current smoker | 61103 | 3.6 | 1.17 (1.12, 1.23) | | <0.001 | 1.07 (1.02, 1.12) | 0.007 |
| Prefer not to  answer/Missing | 24533 | 4.1 | 1.12 (1.05, 1.20) | | 0.001 | 0.99 (0.92, 1.06) | 0.772 |
| Alcohol status |  |  |  |  |  |  |  |
| Never drinker | 52661 | 3.3 | 1 (ref) | | - | 1 (ref) | - |
| Former drinker | 47160 | 3.8 | 0.76 (0.71, 0.82) | | <0.001 | 0.75 (0.70, 0.80) | <0.001 |
| Current drinker | 829643 | 4.7 | 0.98 (0.93, 1.03) | | 0.420 | 1.00 (0.95, 1.06) | 0.903 |
| Prefer not to  answer/Missing | 3652 | 4.7 | 1.14 (0.97, 1.35) | | 0.112 | 1.00 (0.83, 1.19) | 0.967 |
| Family history of CVD |  |  |  |  |  |  |  |
| No | 623580 | 3.8 | 1 (ref) | | - | 1 (ref) | - |
| Yes | 309536 | 6.2 | 1.16 (1.14, 1.18) | | <0.001 | 1.19 (1.17, 1.22) | <0.001 |
| Prior CVD |  |  |  |  |  |  |  |
| No | 919698 | 4.5 | 1 (ref) | | - | 1 (ref) | - |
| Yes | 13418 | 8.0 | 0.95 (0.89, 1.01) | | 0.094 | 0.88 (0.82, 0.94) | <0.001 |
| Diabetes |  |  |  |  |  |  |  |
| No | 917936 | 4.5 | 1 (ref) | | - | 1 (ref) | - |
| Yes | 15180 | 7.4 | 1.08 (1.01, 1.15) | | 0.025 | 0.83 (0.78, 0.89) | <0.001 |
| Frequency of falls |  |  |  |  |  |  |  |
| 0 | 796039 | 4.4 | 1 (ref) | | - | 1 (ref) | - |
| 1 | 88794 | 5.5 | 1.02 (0.99, 1.06) | | 0.177 | 0.94 (0.91, 0.97) | <0.001 |
| ≥2 | 43403 | 5.1 | 1.02 (0.97, 1.07) | | 0.429 | 0.83 (0.79, 0.87) | <0.001 |
| Prefer not to  answer/Missing | 4880 | 3.9 | 1.18 (1.02, 1.37) | | 0.029 | 0.94 (0.80, 1.11) | 0.468 |
| Season at the time of enrolment | | |  |  |  |  |  |
| Winter | 229772 | 5.5 | 1 (ref) | | - | 1 (ref) | - |
| Spring | 246644 | 4.3 | 0.75 (0.73, 0.77) | | <0.001 | 0.74 (0.72, 0.76) | <0.001 |
| Summer | 228767 | 3.6 | 0.62 (0.60, 0.64) | | <0.001 | 0.60 (0.59, 0.62) | <0.001 |
| Autumn | 227933 | 4.9 | 0.85 (0.83, 0.88) | | <0.001 | 0.87 (0.84, 0.89) | <0.001 |

**^1^**: A univariate logistic regression model for each covariate (adjusted for age and sex).

**^2^**: A multivariate logistic regression model including all the covariates in Table 1 except ‘Number of blood pressure lowering medications’ due to its high correlation with the outcome/other covariate.

**^3^**: “Other/Intersex” and “Prefer not to answer” were omitted here due to small percentages (<0.1%)

## Table S7: Logistic regression models identifying factors associated with hypertension (SBP in [130-140] vs <130 mmHg (reference)) among participants who did not report BP lowering medication, excluding those with mean SBP >140 mmHg (N= 899,124)

|  |  |  | **Age and sex adjusted^1^** | | | **Multivariable adjusted^2^** | |
| --- | --- | --- | --- | --- | --- | --- | --- |
| **Covariate** | **N** | **Hypertension, %** | **OR (95%CI)** | | **P value** | **OR (95%CI)** | **P value** |
| Age, year |  |  |  | |  |  |  |
| 18-39 | 283476 | 16.6 | 1 (ref) | | - | 1 (ref) | - |
| 40-49 | 174544 | 19.9 | 1.24 (1.22, 1.26) | | <0.001 | 1.20 (1.18, 1.22) | <0.001 |
| 50-59 | 197068 | 26.1 | 1.82 (1.79, 1.85) | | <0.001 | 1.66 (1.63, 1.68) | <0.001 |
| 60-69 | 168146 | 32.8 | 2.47 (2.44, 2.51) | | <0.001 | 2.26 (2.22, 2.29) | <0.001 |
| 70-79 | 68041 | 40.0 | 3.31 (3.25, 3.37) | | <0.001 | 3.11 (3.05, 3.18) | <0.001 |
| 80+ | 7802 | 45.6 | 3.85 (3.67, 4.03) | | <0.001 | 3.76 (3.58, 3.94) | <0.001 |
| Sex |  |  |  |  |  |  |  |
| Female | 556871 | 17.5 | 1 (ref) | | - | 1 (ref) | - |
| Male | 341690 | 35.5 | 2.60 (2.57, 2.63) | | <0.001 | 2.65 (2.62, 2.68) | <0.001 |
|  |  |  |  | |  |  |  |
| BMI |  |  |  | |  |  |  |
| Underweight /Normal | 397107 | 17.0 | 1 (ref) | | - | 1 (ref) | - |
| Overweight | 328387 | 28.0 | 1.64 (1.62, 1.66) | | <0.001 | 1.63 (1.61, 1.65) | <0.001 |
| Obese | 171967 | 34.3 | 2.75 (2.71, 2.79) | | <0.001 | 2.70 (2.66, 2.73) | <0.001 |
| Ethnicity |  |  |  |  |  |  |  |
| White | 789067 | 25.2 | 1(ref) | | - | 1(ref) | - |
| Black | 14611 | 25.8 | 1.23 (1.18, 1.28) | | <0.001 | 1.11 (1.07, 1.16) | <0.001 |
| South Asian | 37463 | 17.5 | 0.68 (0.66, 0.70) | | <0.001 | 0.71 (0.69, 0.73) | <0.001 |
| East Asian | 13213 | 14.5 | 0.58 (0.55, 0.61) | | <0.001 | 0.74 (0.71, 0.78) | <0.001 |
| Other | 44769 | 17.6 | 0.76 (0.74, 0.78) | | <0.001 | 0.78 (0.76, 0.80) | <0.001 |
| Household income, GBP |  |  |  |  |  |  |  |
| ≥ 100,000 | 135833 | 20.7 | 0.81 (0.79, 0.82) | | <0.001 | 0.87 (0.86, 0.89) | <0.001 |
| 52,000 to 100,000 | 258192 | 23.1 | 0.95 (0.94, 0.97) | | <0.001 | 0.97 (0.95, 0.98) | <0.001 |
| 31,000 to 51,999 | 177243 | 25.7 | 1 (ref) | | - | 1 (ref) | - |
| 18,000 to 30,999 | 118318 | 27.3 | 1.03 (1.01, 1.05) | | 0.002 | 1.02 (1.00, 1.03) | 0.096 |
| < 18,000 | 71804 | 25.9 | 1.01 (0.99, 1.03) | | 0.462 | 0.99 (0.96, 1.01) | 0.168 |
| Prefer not to  answer/Missing | 137734 | 25.3 | 1.00 (0.99, 1.02) | | 0.603 | 1.03 (1.01, 1.04) | 0.005 |
| Education |  |  |  |  |  |  |  |
| Tertiary | 622396 | 22.6 | 1 (ref) | | - | 1 (ref) | - |
| Post-secondary  non- tertiary | 46297 | 29.3 | 1.16 (1.14, 1.19) | | <0.001 | 1.10 (1.08, 1.13) | <0.001 |
| Secondary | 187338 | 27.4 | 1.20 (1.19, 1.22) | | <0.001 | 1.13 (1.11, 1.14) | <0.001 |
| None of the above | 30309 | 33.7 | 1.27 (1.24, 1.31) | | <0.001 | 1.15 (1.12, 1.18) | <0.001 |
| Prefer not to  answer/Missing | 12784 | 26.7 | 1.15 (1.11, 1.20) | | <0.001 | 1.09 (1.05, 1.14) | <0.001 |
| Smoking status |  |  |  |  |  |  |  |
| Never smoker | 591045 | 23.4 | 1 (ref) | | - | 1 (ref) | - |
| Former smoker | 224913 | 27.2 | 1.07 (1.06, 1.08) | | <0.001 | 0.98 (0.97, 0.99) | 0.002 |
| Current smoker | 59419 | 23.7 | 1.07 (1.05, 1.10) | | <0.001 | 0.99 (0.97, 1.01) | 0.285 |
| Prefer not to  answer/Missing | 23747 | 24.0 | 1.06 (1.02, 1.09) | | 0.001 | 0.99 (0.96, 1.02) | 0.480 |
| Alcohol status |  |  |  |  |  |  |  |
| Never drinker | 51267 | 19.2 | 1 (ref) | | - | 1 (ref) | - |
| Former drinker | 45803 | 22.1 | 0.99 (0.96, 1.02) | | 0.622 | 0.91 (0.88, 0.94) | <0.001 |
| Current drinker | 798537 | 24.8 | 1.17 (1.15, 1.20) | | <0.001 | 1.10 (1.08, 1.13) | <0.001 |
| Prefer not to  answer/Missing | 3517 | 24.4 | 1.12 (1.03, 1.21) | | 0.010 | 1.03 (0.94, 1.12) | 0.586 |
| Family history of CVD |  |  |  |  |  |  |  |
| No | 605380 | 22.8 | 1 (ref) | | - | 1 (ref) | - |
| Yes | 293744 | 27.6 | 1.12 (1.11, 1.13) | | <0.001 | 1.12 (1.11, 1.14) | <0.001 |
| Prior CVD |  |  |  |  |  |  |  |
| No | 886622 | 24.2 | 1 (ref) | | - | 1 (ref) | - |
| Yes | 12502 | 32.0 | 1.00 (0.96, 1.04) | | 0.912 | 0.94 (0.91, 0.98) | 0.005 |
| Diabetes |  |  |  |  |  |  |  |
| No | 884864 | 24.2 | 1 (ref) | | - | 1 (ref) | - |
| Yes | 14260 | 33.0 | 1.20 (1.15, 1.24) | | <0.001 | 1.01 (0.97, 1.05) | 0.526 |
| Frequency of falls |  |  |  |  |  |  |  |
| 0 | 768021 | 24.2 | 1 (ref) | | - | 1 (ref) | - |
| 1 | 84769 | 25.3 | 1.04 (1.02, 1.06) | | <0.001 | 0.98 (0.97, 1.00) | 0.054 |
| ≥2 | 41602 | 25.5 | 1.09 (1.07, 1.12) | | <0.001 | 0.95 (0.93, 0.98) | <0.001 |
| Prefer not to  answer/Missing | 4732 | 22.4 | 0.98 (0.91, 1.05) | | 0.557 | 0.95 (0.88, 1.03) | 0.202 |
| Season at the time of enrolment | | |  |  |  |  |  |
| Winter | 219447 | 25.7 | 1 (ref) | | - | 1 (ref) | - |
| Spring | 238286 | 23.6 | 0.88 (0.87, 0.89) | | <0.001 | 0.87 (0.86, 0.88) | <0.001 |
| Summer | 222443 | 22.7 | 0.82 (0.80, 0.83) | | <0.001 | 0.80 (0.79, 0.81) | <0.001 |
| Autumn | 218948 | 25.5 | 0.97 (0.95, 0.98) | | <0.001 | 0.97 (0.96, 0.99) | <0.001 |

**^1^**: A univariate logistic regression model for each covariate (adjusted for age and sex).

**^2^**: A multivariate logistic regression model including all the covariates in Table 1 except ‘Number of blood pressure lowering medications’ due to its high correlation with the outcome/other covariate.

**^3^**: “Other/Intersex” and “Prefer not to answer” were omitted here due to small percentages (<0.1%)

## Table S8: Logistic regression models identifying factors associated with hypertension (BP >=140/90 vs. <140/90 mm Hg (reference)) among study population with detected hypertension (N= 212,431)

|  |  |  | **Age and sex adjusted^1^** | | | **Multivariable adjusted^2^** | |
| --- | --- | --- | --- | --- | --- | --- | --- |
| **Covariate** | **N** | **Hypertension, %** | **OR (95%CI)** | | **P value** | **OR (95%CI)** | **P value** |
| Age, year |  |  |  | |  |  |  |
| 18-39 | 6782 | 48.2 | 1 (ref) | | - | 1 (ref) | - |
| 40-49 | 14439 | 56.4 | 1.38 (1.30, 1.46) | | <0.001 | 1.46 (1.38, 1.55) | <0.001 |
| 50-59 | 41644 | 53.9 | 1.24 (1.17, 1.30) | | <0.001 | 1.35 (1.28, 1.43) | <0.001 |
| 60-69 | 75560 | 50.5 | 1.07 (1.02, 1.13) | | 0.007 | 1.25 (1.19, 1.32) | <0.001 |
| 70-79 | 62282 | 50.8 | 1.08 (1.03, 1.14) | | 0.002 | 1.38 (1.31, 1.46) | <0.001 |
| 80+ | 11652 | 53.7 | 1.21 (1.14, 1.29) | | <0.001 | 1.73 (1.63, 1.85) | <0.001 |
| Sex |  |  |  |  |  |  |  |
| Female | 93170 | 48.8 | 1 (ref) | | - | 1 (ref) | - |
| Male | 119171 | 54.1 | 1.24 (1.22, 1.26) | | <0.001 | 1.33 (1.30, 1.35) | <0.001 |
| BMI |  |  |  | |  |  |  |
| Underweight /Normal | 43565 | 44.0 | 1 (ref) | | - | 1 (ref) | - |
| Overweight | 85206 | 49.7 | 1.22 (1.19, 1.25) | | <0.001 | 1.23 (1.21, 1.26) | <0.001 |
| Obese | 83155 | 58.0 | 1.73 (1.69, 1.77) | | <0.001 | 1.83 (1.79, 1.88) | <0.001 |
| Ethnicity |  |  |  |  |  |  |  |
| White | 196610 | 52.0 | 1(ref) | | - | 1(ref) | - |
| Black | 3650 | 56.8 | 1.18 (1.10, 1.26) | | <0.001 | 1.13 (1.06, 1.21) | <0.001 |
| South Asian | 5360 | 46.0 | 0.74 (0.70, 0.78) | | <0.001 | 0.85 (0.80, 0.90) | <0.001 |
| East Asian | 1366 | 44.6 | 0.72 (0.64, 0.80) | | <0.001 | 0.81 (0.73, 0.90) | <0.001 |
| Other | 5445 | 47.3 | 0.80 (0.76, 0.85) | | <0.001 | 0.84 (0.80, 0.89) | <0.001 |
| Household income, GBP |  |  |  |  |  |  |  |
| ≥ 100,000 | 15873 | 50.6 | 0.86 (0.83, 0.90) | | <0.001 | 0.87 (0.84, 0.91) | <0.001 |
| 52,000 to 99,999 | 41849 | 52.3 | 0.96 (0.94, 0.99) | | 0.006 | 0.96 (0.94, 0.99) | 0.006 |
| 31,000 to 51,999 | 48345 | 52.3 | 1 (ref) | | - | 1 (ref) | - |
| 18,000 to 30,999 | 43194 | 51.8 | 1.00 (0.98, 1.03) | | 0.928 | 1.00 (0.97, 1.02) | 0.806 |
| < 18,000 | 26240 | 50.3 | 0.95 (0.93, 0.98) | | 0.003 | 0.95 (0.92, 0.98) | 0.002 |
| Prefer not to  answer/Missing | 36930 | 52.3 | 1.03 (1.00, 1.06) | | 0.046 | 1.02 (0.99, 1.05) | 0.122 |
| Education |  |  |  |  |  |  |  |
| Tertiary | 121013 | 51.3 | 1 (ref) | | - | 1 (ref) | - |
| Post-secondary  non- tertiary | 18733 | 51.4 | 1.03 (1.00, 1.07) | | 0.030 | 1.02 (0.99, 1.05) | 0.184 |
| Secondary | 53243 | 52.5 | 1.07 (1.05, 1.09) | | <0.001 | 1.05 (1.02, 1.07) | <0.001 |
| None of the above | 16514 | 53.2 | 1.11 (1.07, 1.15) | | <0.001 | 1.08 (1.05, 1.12) | <0.001 |
| Prefer not to  answer/Missing | 2928 | 54.4 | 1.14 (1.06, 1.23) | | <0.001 | 1.10 (1.02, 1.19) | 0.014 |
| Smoking status |  |  |  |  |  |  |  |
| Never smoker | 121270 | 51.7 | 1 (ref) | | - | 1 (ref) | - |
| Former smoker | 76822 | 51.9 | 0.99 (0.98, 1.01) | | 0.534 | 0.98 (0.96, 1.00) | 0.013 |
| Current smoker | 9886 | 51.9 | 0.97 (0.93, 1.01) | | 0.205 | 1.01 (0.96, 1.05) | 0.783 |
| Prefer not to  answer/Missing | 4453 | 51.6 | 0.95 (0.90, 1.01) | | 0.123 | 0.94 (0.89, 1.00) | 0.069 |
| Alcohol status |  |  |  |  |  |  |  |
| Never drinker | 8958 | 48.3 | 1 (ref) | | - | 1 (ref) | - |
| Former drinker | 12575 | 47.1 | 0.94 (0.89, 1.00) | | 0.039 | 0.92 (0.87, 0.97) | 0.004 |
| Current drinker | 190380 | 52.3 | 1.15 (1.11, 1.20) | | 0.000 | 1.09 (1.04, 1.14) | <0.001 |
| Prefer not to  answer/Missing | 518 | 50.2 | 1.05 (0.88, 1.25) | | 0.594 | 0.97 (0.81, 1.17) | 0.762 |
| Family history of CVD |  |  |  |  |  |  |  |
| No | 95374 | 52.8 | 1 (ref) | | - | 1 (ref) | - |
| Yes | 117057 | 51.0 | 0.96 (0.94, 0.97) | | <0.001 | 1.00 (0.99, 1.02) | 0.733 |
| Prior CVD |  |  |  |  |  |  |  |
| No | 173905 | 54.2 | 1 (ref) | | - | 1 (ref) | - |
| Yes | 38526 | 40.7 | 0.55 (0.54, 0.57) | | <0.001 | 0.62 (0.60, 0.63) | <0.001 |
| Diabetes |  |  |  |  |  |  |  |
| No | 194826 | 52.1 | 1 (ref) | | - | 1 (ref) | - |
| Yes | 17605 | 48.2 | 0.84 (0.82, 0.87) | | <0.001 | 0.81 (0.78, 0.83) | <0.001 |
| Frequency of falls |  |  |  |  |  |  |  |
| 0 | 166252 | 52.3 | 1 (ref) | | - | 1 (ref) | - |
| 1 | 28263 | 50.6 | 0.96 (0.94, 0.99) | | 0.004 | 0.94 (0.92, 0.97) | <0.001 |
| ≥ 2 | 17279 | 48.9 | 0.90 (0.87, 0.93) | | <0.001 | 0.90 (0.87, 0.93) | <0.001 |
| Prefer not to  answer/Missing | 637 | 49.5 | 0.90 (0.77, 1.05) | | 0.194 | 0.87 (0.74, 1.02) | 0.094 |
| Season at the time of enrolment | | |  |  |  |  |  |
| Winter | 52780 | 55.5 | 1 (ref) | | - | 1 (ref) | - |
| Spring | 55710 | 49.9 | 0.80 (0.78, 0.82) | | <0.001 | 0.79 (0.77, 0.81) | <0.001 |
| Summer | 50447 | 47.3 | 0.72 (0.70, 0.74) | | <0.001 | 0.71 (0.69, 0.73) | <0.001 |
| Autumn | 53494 | 54.2 | 0.95 (0.93, 0.97) | | <0.001 | 0.93 (0.90, 0.95) | <0.001 |
| Number of BP lowering medication | |  |  | |  |  |  |
| 0 | 27655 | 61.4 | 1 (ref) | | - | 1 (ref) | - |
| 1 | 142714 | 51.8 | 0.66 (0.64, 0.68) | | <0.001 | 0.65 (0.63, 0.67) | <0.001 |
| 2 | 30135 | 43.0 | 0.46 (0.44, 0.47) | | <0.001 | 0.49 (0.48, 0.51) | <0.001 |
| >=3 | 4243 | 39.1 | 0.39 (0.36, 0.41) | | <0.001 | 0.42 (0.39, 0.45) | <0.001 |
| Missing | 7684 | 58.0 | 0.85 (0.81, 0.89) | | <0.001 | 0.80 (0.75, 0.84) | <0.001 |

**^1^**: A univariate logistic regression model for each covariate (adjusted for age and sex).

**^2^**: A multivariate logistic regression model including all the covariates.

**^3^**: “Other/Intersex” and “Prefer not to answer” were omitted here due to small percentages (<0.1%)

## Table S9: Logistic regression models identifying factors associated with indicator of falls (Yes vs. No (reference)) among study population with detected hypertension (N= 212,431)

|  |  |  | **Age and sex adjusted^1^** | | | **Multivariable adjusted^2^** | |
| --- | --- | --- | --- | --- | --- | --- | --- |
| **Covariate** | **N** | **Falls, %** | **OR (95%CI)** | | **P value** | **OR (95%CI)** | **P value** |
| Age, year |  |  |  | |  |  |  |
| 18-39 | 6726 | 19.0 | 1 (ref) | | - | 1 (ref) | - |
| 40-49 | 14365 | 17.0 | 0.90 (0.83, 0.97) | | 0.005 | 0.91 (0.85, 0.99) | 0.020 |
| 50-59 | 41500 | 19.1 | 1.05 (0.98, 1.12) | | 0.177 | 1.06 (0.99, 1.14) | 0.085 |
| 60-69 | 75345 | 19.9 | 1.14 (1.07, 1.21) | | <0.001 | 1.09 (1.02, 1.17) | 0.008 |
| 70-79 | 62151 | 24.1 | 1.46 (1.37, 1.56) | | <0.001 | 1.41 (1.32, 1.51) | <0.001 |
| 80+ | 11635 | 33.3 | 2.32 (2.16, 2.50) | | <0.001 | 2.29 (2.12, 2.47) | <0.001 |
| Sex |  |  |  |  |  |  |  |
| Female | 92854 | 26.4 | 1 (ref) | | - | 1 (ref) | - |
| Male | 118850 | 17.6 | 0.58 (0.57, 0.60) | | <0.001 | 0.59 (0.58, 0.60) | <0.001 |
| Prefer not to answer | 74 | 32.4 | 1.34 (0.82, 2.19) | | 0.235 | 1.26 (0.76, 2.09) | 0.365 |
| BMI |  |  |  | |  |  |  |
| Underweight /Normal | 43476 | 19.4 | 1 (ref) | | - | 1 (ref) | - |
| Overweight | 84980 | 19.3 | 1.13 (1.10, 1.17) | | <0.001 | 1.11 (1.08, 1.15) | <0.001 |
| Obese | 82835 | 24.7 | 1.62 (1.58, 1.67) | | <0.001 | 1.50 (1.45, 1.55) | <0.001 |
| Ethnicity |  |  |  |  |  |  |  |
| White | 196095 | 21.8 | 1(ref) | | - | 1(ref) | - |
| Black | 3630 | 17.8 | 0.84 (0.77, 0.91) | | <0.001 | 0.76 (0.70, 0.83) | <0.001 |
| South Asian | 5316 | 16.6 | 0.83 (0.77, 0.89) | | <0.001 | 0.79 (0.73, 0.85) | <0.001 |
| East Asian | 1355 | 15.9 | 0.74 (0.64, 0.86) | | <0.001 | 0.82 (0.70, 0.95) | 0.008 |
| Other | 5398 | 20.4 | 0.99 (0.93, 1.06) | | 0.837 | 0.93 (0.87, 1.00) | 0.040 |
| Household income, GBP |  |  |  |  |  |  |  |
| ≥ 100,000 | 15862 | 12.4 | 0.65 (0.61, 0.68) | | <0.001 | 0.70 (0.66, 0.74) | <0.001 |
| 52,000 to 100,000 | 41810 | 16.1 | 0.82 (0.79, 0.85) | | <0.001 | 0.84 (0.81, 0.87) | <0.001 |
| 31,000 to 51,999 | 48282 | 19.9 | 1 (ref) | | - | 1 (ref) | - |
| 18,000 to 30,999 | 43098 | 24.3 | 1.23 (1.19, 1.27) | | <0.001 | 1.19 (1.15, 1.23) | <0.001 |
| < 18,000 | 26109 | 31.5 | 1.72 (1.66, 1.78) | | <0.001 | 1.57 (1.52, 1.63) | 0.39 |
| Prefer not to  answer/Missing | 36633 | 23.3 | 1.14 (1.11, 1.18) | | <0.001 | 1.13 (1.09, 1.17) | 0.07 |
| Education |  |  |  |  |  |  |  |
| Tertiary | 120756 | 20.3 | 1 (ref) | | - | 1 (ref) | - |
| Post-secondary  non- tertiary | 18688 | 22.6 | 1.03 (0.99, 1.07) | | 0.146 | 0.98 (0.94, 1.01) | 0.216 |
| Secondary | 53099 | 21.9 | 1.04 (1.02, 1.07) | | 0.001 | 0.94 (0.91, 0.96) | 0.000 |
| None of the above | 16409 | 27.0 | 1.26 (1.21, 1.31) | | <0.001 | 0.99 (0.95, 1.03) | 0.610 |
| Prefer not to  answer/Missing | 2842 | 23.5 | 1.14 (1.04, 1.25) | | 0.004 | 0.94 (0.86, 1.03) | 0.182 |
| Smoking status |  |  |  |  |  |  |  |
| Never smoker | 120948 | 20.1 | 1 (ref) | | - | 1 (ref) | - |
| Former smoker | 76636 | 22.7 | 1.19 (1.16, 1.22) | | <0.001 | 1.11 (1.09, 1.14) | <0.001 |
| Current smoker | 9822 | 28.1 | 1.80 (1.72, 1.89) | | <0.001 | 1.58 (1.50, 1.66) | <0.001 |
| Prefer not to  answer/Missing | 4388 | 23.5 | 1.34 (1.25, 1.44) | | <0.001 | 1.24 (1.15, 1.33) | <0.001 |
| Alcohol status |  |  |  |  |  |  |  |
| Never drinker | 8866 | 25.4 | 1 (ref) | | - | 1 (ref) | - |
| Former drinker | 12516 | 27.3 | 1.18 (1.11, 1.26) | | 1.18 (1.11, 1.26) | 1.06 (1.00, 1.14) | 0.064 |
| Current drinker | 189934 | 20.9 | 0.83 (0.79, 0.88) | | 0.83 (0.79, 0.88) | 0.85 (0.81, 0.90) | <0.001 |
| Prefer not to  answer/Missing | 478 | 28.7 | 1.33 (1.08, 1.64) | | 0.007 | 1.19 (0.96, 1.47) | 0.106 |
| Family history of CVD |  |  |  |  |  |  |  |
| No | 95045 | 20.3 | 1 (ref) | | - | 1 (ref) | - |
| Yes | 116749 | 22.5 | 1.08 (1.06, 1.11) | | <0.001 | 1.08 (1.06, 1.11) | <0.001 |
| Prior CVD |  |  |  |  |  |  |  |
| No | 173411 | 20.6 | 1 (ref) | | - | 1 (ref) | - |
| Yes | 38383 | 25.4 | 1.36 (1.32, 1.40) | | <0.001 | 1.27 (1.24, 1.31) | <0.001 |
| Diabetes |  |  |  |  |  |  |  |
| No | 194265 | 20.7 | 1 (ref) | | - | 1 (ref) | - |
| Yes | 17529 | 30.5 | 1.77 (1.71, 1.83) | | <0.001 | 1.54 (1.49, 1.59) | <0.001 |
| Season at the time of enrolment | | |  |  |  |  |  |
| Winter | 52624 | 21.4 | 1 (ref) | | - | 1 (ref) | - |
| Spring | 55530 | 21.3 | 0.99 (0.96, 1.02) | | 0.586 | 0.99 (0.96, 1.02) | 0.444 |
| Summer | 50296 | 22.1 | 1.04 (1.01, 1.07) | | 0.010 | 1.03 (1.00, 1.06) | 0.044 |
| Autumn | 53344 | 21.2 | 0.99 (0.96, 1.02) | | 0.482 | 0.99 (0.96, 1.03) | 0.701 |
| Number of BP lowering medication | |  |  | |  |  |  |
| 0 | 27520 | 23.5 | 1 (ref) | | - | 1 (ref) | - |
| 1 | 142322 | 20.5 | 0.82 (0.79, 0.84) | | 0.000 | 0.82 (0.79, 0.84) | <0.001 |
| 2 | 30055 | 23.9 | 1.00 (0.96, 1.04) | | 0.805 | 0.87 (0.84, 0.91) | <0.001 |
| >=3 | 4230 | 28.1 | 1.19 (1.10, 1.28) | | 0.000 | 0.97 (0.90, 1.05) | 0.488 |
| Missing | 7667 | 20.2 | 0.83 (0.78, 0.88) | | 0.000 | 0.79 (0.74, 0.85) | <0.001 |

**^1^**: A univariate logistic regression model for each covariate (adjusted for age and sex).

**^2^**: A multivariate logistic regression model including all the covariates in Table 1 except ‘Frequency of fall’ due to its high correlation with the outcome.

**^3^**: “Other/Intersex” and “Prefer not to answer” were omitted here due to small percentages (<0.1%)

# Supplementary analysis including participants with serious health conditions (kidney failure, heart failure, liver failure, and cancer other than basal/squamous cell skin cancer)

## Figure S2. Study flowchart including participants with serious health conditions

**Figure S2.** Study flowchart including participants with serious health conditions. “**Hypertension**” was defined based on the WHO guidelines as the number of participants who (i) had mean SBP of ≥140 mmHg or mean DBP ≥ 90 mmHg, or (ii) were receiving antihypertensive medications. “**Detected**” was defined as report of a prior diagnosis of hypertension by a health professional or receiving antihypertensive medication, among those who were hypertensive. “**Treated**” was defined as report of hypertensive medication. “**Controlled**” was defined if SBP was <140 mmHg and DBP <90 mmHg among those taking antihypertensive medication. OFH: Our Future Health; BP: blood pressure; SBP: systolic blood pressure; DBP: diastolic blood pressure; WHO: World Health Organization.

## Figure S3. Prevalence of hypertension by age and sex

***Figure S3****. Prevalence of hypertension by age and sex, defined using WHO criteria, with crude prevalence for all age groups by sex denoted as “All” and age-standardised prevalence as “All (std)”. Direct standardisation was based on the* [ONS](https://www.ons.gov.uk/peoplepopulationandcommunity/populationandmigration/populationestimates/datasets/populationestimatesforukenglandandwalesscotlandandnorthernireland) *population estimates for England in mid-2023.*

## Table S10: Associations of risk factors with hypertension defined using WHO criteria (Yes vs. No [reference]) in the study population (N=1,428,082)

|  |  |  | **Age and sex adjusted^1^** | | | **Multivariable adjusted^2^** | |
| --- | --- | --- | --- | --- | --- | --- | --- |
| **Covariates** | **N** | **Hypertension, %** | **OR (95%CI)** | | **P value** | **OR (95%CI)** | **P value** |
| Age, year |  |  |  | |  |  |  |
| 18-39 | 315476 | 15.4 | 1 (ref) | | - | 1 (ref) | - |
| 40-49 | 217362 | 25.7 | 1.91 (1.88, 1.94) | | <0.001 | 1.76 (1.73, 1.79) | <0.001 |
| 50-59 | 304639 | 38.2 | 3.47 (3.43, 3.51) | | <0.001 | 2.92 (2.88, 2.95) | <0.001 |
| 60-69 | 347879 | 50.2 | 5.54 (5.47, 5.60) | | <0.001 | 4.53 (4.47, 4.59) | <0.001 |
| 70-79 | 208320 | 62.7 | 9.08 (8.96, 9.20) | | <0.001 | 7.64 (7.53, 7.75) | <0.001 |
| 80+ | 34207 | 71.6 | 13.14 (12.80, 13.48) | | <0.001 | 11.43 (11.12, 11.75) | <0.001 |
| Sex |  |  |  |  |  |  |  |
| Female | 796596 | 30.6 | 1 (ref) | | - | 1 (ref) | - |
| Male | 630681 | 48.6 | 2.08 (2.07, 2.10) | | <0.001 | 2.02 (2.00, 2.03) | <0.001 |
|  |  |  |  | |  |  |  |
| BMI |  |  |  | |  |  |  |
| Underweight /Normal | 530368 | 23.7 | 1 (ref) | | - | 1 (ref) | - |
| Overweight | 543617 | 41.1 | 1.92 (1.90, 1.94) | | <0.001 | 1.88 (1.86, 1.90) | <0.001 |
| Obese | 351358 | 57.0 | 4.72 (4.67, 4.77) | | <0.001 | 4.44 (4.40, 4.49) | <0.001 |
| Ethnicity |  |  |  |  |  |  |  |
| White | 1283267 | 39.7 | 1(ref) | | - | 1(ref) | - |
| Black | 22371 | 39.5 | 1.55 (1.50, 1.59) | | <0.001 | 1.32 (1.28, 1.36) | <0.001 |
| South Asian | 48315 | 27.8 | 0.88 (0.86, 0.90) | | <0.001 | 0.93 (0.91, 0.95) | <0.001 |
| East Asian | 16453 | 22.8 | 0.65 (0.63, 0.68) | | <0.001 | 0.99 (0.95, 1.03) | 0.70 |
| Other | 57675 | 26 | 0.84 (0.82, 0.85) | | <0.001 | 0.86 (0.84, 0.88) | <0.001 |
| Household income, GBP |  |  |  |  |  |  |  |
| ≥ 100,000 | 180101 | 27 | 0.68 (0.68, 0.69) | | <0.001 | 0.79 (0.78, 0.80) | <0.001 |
| 52,000 to 100,000 | 370846 | 33 | 0.88 (0.87, 0.89) | | <0.001 | 0.92 (0.91, 0.93) | <0.001 |
| 31,000 to 51,999 | 296165 | 41.5 | 1 (ref) | | - | 1 (ref) | - |
| 18,000 to 30,999 | 218718 | 46.4 | 1.08 (1.07, 1.10) | | <0.001 | 1.03 (1.01, 1.04) | <0.001 |
| ≤ 18,000 | 131084 | 45.7 | 1.12 (1.11, 1.14) | | <0.001 | 1.00 (0.99, 1.02) | 0.91 |
| Prefer not to  answer/Missing | 231168 | 41.2 | 0.99 (0.98, 1.01) | | 0.30 | 0.99 (0.98, 1.00) | 0.08 |
| Education |  |  |  |  |  |  |  |
| Tertiary | 926887 | 34.7 | 1 (ref) | | - | 1 (ref) | - |
| Post-secondary  non- tertiary | 90343 | 48.2 | 1.18 (1.16, 1.19) | | <0.001 | 1.09 (1.08, 1.11) | <0.001 |
| Secondary | 321318 | 43 | 1.22 (1.21, 1.23) | | <0.001 | 1.12 (1.10, 1.13) | <0.001 |
| None of the above | 68801 | 55.7 | 1.38 (1.35, 1.40) | | <0.001 | 1.14 (1.12, 1.16) | <0.001 |
| Prefer not to  answer/Missing | 20733 | 41.6 | 1.24 (1.20, 1.28) | | <0.001 | 1.11 (1.07, 1.14) | <0.001 |
| Smoking status |  |  |  |  |  |  |  |
| Never smoker | 902769 | 36.1 | 1 (ref) | | - | 1 (ref) | - |
| Former smoker | 405321 | 45.1 | 1.15 (1.14, 1.16) | | <0.001 | 1.02 (1.01, 1.03) | <0.001 |
| Current smoker | 84529 | 34.4 | 1.18 (1.17, 1.20) | | <0.001 | 1.06 (1.04, 1.08) | <0.001 |
| Prefer not to  answer/Missing | 35463 | 36.7 | 1.12 (1.09, 1.15) | | <0.001 | 1.00 (0.98, 1.03) | 0.84 |
| Alcohol status |  |  |  |  |  |  |  |
| Never drinker | 72405 | 31.9 | 1 (ref) | | - | 1 (ref) | - |
| Former drinker | 74834 | 38.4 | 0.97 (0.95, 0.99) | | 0.01 | 0.93 (0.91, 0.96) | <0.001 |
| Current drinker | 1275728 | 39 | 1.00 (0.99, 1.02) | | 0.76 | 1.08 (1.06, 1.10) | <0.001 |
| Prefer not to  answer/Missing | 5115 | 35.9 | 1.02 (0.96, 1.09) | | 0.52 | 1.04 (0.97, 1.12) | 0.28 |
| Family history of CVD |  |  |  |  |  |  |  |
| No | 880881 | 33.3 | 1 (ref) | | - | 1 (ref) | - |
| Yes | 547201 | 47 | 1.35 (1.34, 1.36) | | <0.001 | 1.30 (1.29, 1.31) | <0.001 |
| Prior CVD |  |  |  |  |  |  |  |
| No | 1362154 | 36.5 | 1 (ref) | | - | 1 (ref) | - |
| Yes | 65928 | 80.2 | 4.13 (4.05, 4.22) | | <0.001 | 3.68 (3.61, 3.76) | <0.001 |
| Diabetes |  |  |  |  |  |  |  |
| No | 1386207 | 37.7 | 1 (ref) | | - | 1 (ref) | - |
| Yes | 41875 | 65.8 | 2.32 (2.27, 2.37) | | <0.001 | 1.57 (1.53, 1.60) | <0.001 |
| Frequency of falls |  |  |  |  |  |  |  |
| 0 | 1194574 | 37.4 | 1 (ref) | | - | 1 (ref) | - |
| 1 | 149587 | 43.7 | 1.13 (1.12, 1.14) | | <0.001 | 1.00 (0.99, 1.02) | 0.64 |
| ≥2 | 77331 | 46.7 | 1.37 (1.34, 1.39) | | <0.001 | 1.00 (0.99, 1.02) | 0.61 |
| Prefer not to  answer/Missing | 6590 | 33 | 1.08 (1.02, 1.14) | | 0.01 | 0.93 (0.87, 0.99) | 0.02 |
| Season at the time of enrolment | | |  |  |  |  |  |
| Winter | 358007 | 40.2 | 1 (ref) | | - | 1 (ref) | - |
| Spring | 375379 | 37.6 | 0.89 (0.88, 0.90) | | <0.001 | 0.87 (0.86, 0.88) | <0.001 |
| Summer | 343980 | 36.7 | 0.84 (0.84, 0.85) | | <0.001 | 0.82 (0.81, 0.83) | <0.001 |
| Autumn | 350716 | 39.7 | 0.96 (0.95, 0.97) | | <0.001 | 0.97 (0.96, 0.98) | <0.001 |

**^1^**: A univariate logistic regression model for each covariate (adjusted for age and sex).

**^2^**: A multivariate logistic regression model including all the covariates in Table 1 except ‘Number of blood pressure lowering medications’ due to its high correlation with the outcome/other covariates.

**^3^**: “Other/Intersex” and “Prefer not to answer” were omitted here due to small percentages (<0.1%)
